# Visualizing electron delocalization in contorted polycyclic aromatic hydrocarbons†

**DOI:** 10.1039/d1sc03368a

**Published:** 2021-09-08

**Authors:** Albert Artigas, Denis Hagebaum-Reignier, Yannick Carissan, Yoann Coquerel

**Affiliations:** Aix Marseille Université, CNRS, Centrale Marseille, ISM2 13397 Marseille France yannick.carissan@univ-amu.fr yoann.coquerel@univ-amu.fr

## Abstract

Electron delocalization in contorted polycyclic aromatic hydrocarbon (PAH) molecules was examined through 3D isotropic magnetic shielding (IMS) contour maps built around the molecules using pseudo-van der Waals surfaces. The resulting maps of electron delocalization provided an intuitive, yet detailed and quantitative evaluation of the aromatic, non aromatic, and antiaromatic character of the local and global conjugated cyclic circuits distributed over the molecules. An attractive pictural feature of the 3D IMS contour maps is that they are reminiscent of the Clar π-sextet model of aromaticity. The difference in delocalization patterns between the two faces of the electron circuits in contorted PAHs was clearly visualized. For π-extended contorted PAHs, some splits of the π system resulted in recognizable patterns typical of smaller PAHs. The differences between the delocalization patterns of diastereomeric chiral PAHs could also be visualized. Mapping IMS on pseudo-van der Waals surfaces around contorted PAHs allowed visualization of their superimposed preferred circuits for electron delocalization and hence their local and global aromaticity patterns.

## Abstract 2

Electron delocalization in contorted polycyclic aromatic hydrocarbon (PAH) molecules was examined through 3D isotropic magnetic shielding (IMS) contour maps built around the molecules using pseudo-van der Waals surfaces.

## Graphical abstract

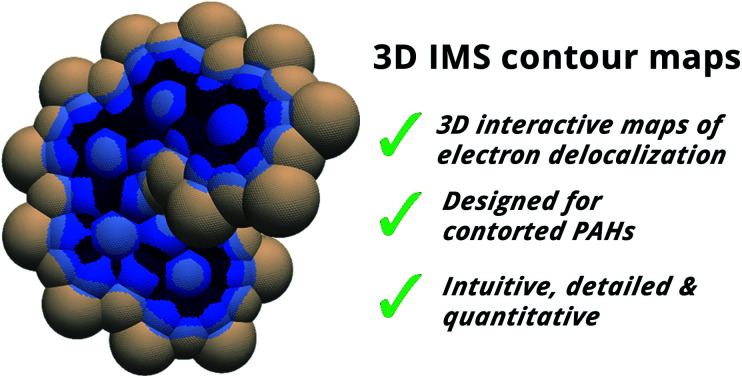

## Introduction

Aromatic compounds have fascinated chemists for nearly two centuries,^1–4^ and the concept of aromaticity has become a topic of primary importance in contemporaneous chemical sciences.^5–16^ Polycyclic aromatic hydrocarbons (PAHs) have for long been considered as a class of planar molecules made of fused hexagons with alternating single and double bonds. With progress of organic synthesis this paradigm has gradually shifted, and a variety of nonplanar PAHs, possibly chiral and/or embedding odd-membered rings, are now available.^17–24^ Contrary to what is occurring in their planar counterparts, the determination of π and σ orbital blocks in nonplanar molecules and the analysis of their magnetic properties is a challenge, especially for the assessment of their local and global aromatic character. To do so, computational methods based on magnetic criteria have emerged as reliable tools and constitute the state-of-the-art approaches.^10,16,25,26^ The physical principle underlying these tools consists in applying an external magnetic field to a molecule and calculating its magnetic response. For planar molecules, anisotropic approaches requiring the definition of a privileged direction for the analysis, typically perpendicular to the molecular plane, are best suited.^25–29^ For contorted molecules, definition of a privileged direction is ambiguous, if not unrealistic, and isotropic approaches averaging the magnetic response in the three directions of space should generally be employed. Adjustments are of course possible.^30–34^

The most popular isotropic approach to assess the magnetic properties of molecules is the isotropic calculation of the nucleus independent chemical shift (NICS), also called NICS_iso_. It reports the magnetic response of a molecule at any location in space (referred to as *Bq* in the following) as the negative of the calculated isotropic magnetic shielding (IMS) value at this location.^35^ NICS calculations have proven of considerable value tough they have some identified limitations. For instance, (a) NICS only reports a number (in ppm) at a defined position in space; it is an indirect method that incurs a loss of information on the magnetic field it senses. (b) The calculation of NICS_iso_ (and IMS) includes significant contributions from the σ system (not relevant to aromaticity) due to its averaged nature and should be interpreted accordingly. This is thoroughly discussed in some excellent reviews.^16,29^

NICS has routinely been computed as single points 1 Å above the middle of each ring.^36,37^ Richer pictures of NICS in molecules can be obtained by mapping NICS using 1D,^38–41^ 2D,^37,42–48^ and 3D grids^49–57^ of *Bq*. The latter approach can produce 3D maps of the magnetic properties of molecules as isovalue surfaces. While isovalue surface representations of IMS offer a suitable approach to study magnetic properties, the 3D mapping of IMS isocontour values at a short distance from contorted PAHs appears as a complementary alternative for the analysis and the exploration of electron delocalization in these classes of systems.

“*Il faut plutôt regarder les choses beaucoup de fois. Et en changeant à chaque fois d'angle, pas deux fois sous le même angle. Les aborder une fois en dessus, une fois en dessous, une fois de biais – surtout de biais.*”

“It is better to observe things many times, each time changing one's point of view, and never at the same angle. Approach now from above, now from below, now obliquely – most of all, look obliquely”, from Causette, 1947 | Jean Dubuffet, painter and sculptor (1901–1985).

**Fig. 1:**
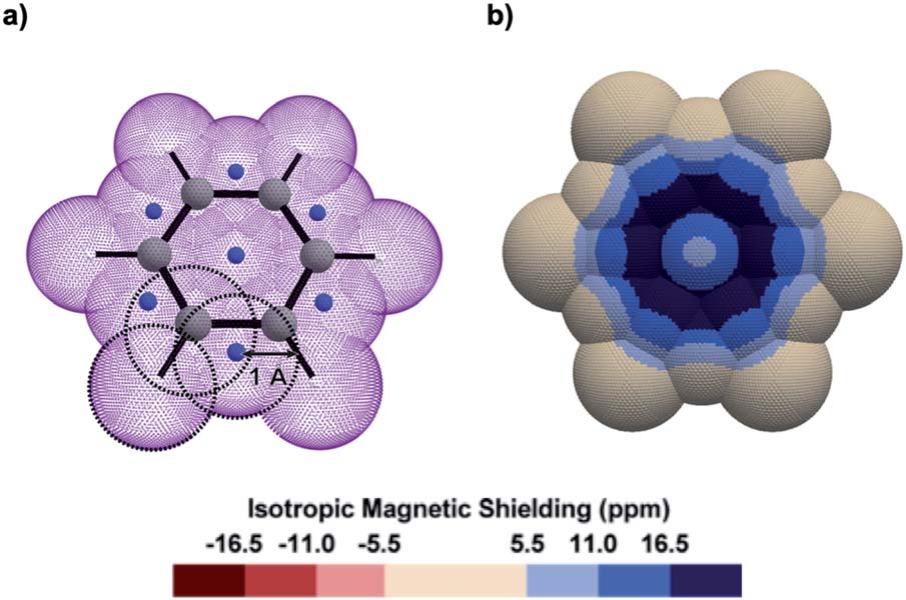
(a) Oblique view of a pseudo-van der Waals surface of *Bq* (purple colour) generated over benzene by the overlap of 19 spheres of 1 Å radius centred on the C (grey colour), H (white colour), and dummy (artificially introduced at specific barycentres as described in Fig. S1 in the ESI,† blue colour) nuclei. (b) Top view of the corresponding 3D IMS contour map of benzene calculated at the B3LYP-GIAO/6-311++G**//B3LYP-D3BJ/def2-SVP level of theory.

Looking obliquely at this problem, we analysed IMS on 3D surfaces generated around molecules using a pseudo-van der Waals approach (Fig. 1a). IMS values were computed at each point on these surfaces and plotted by using a colour code, thus generating 3D contour maps that smoothly follow the molecules' geometry and inform on their magnetic properties. As a paradigmatic example, the 3D IMS contour map of benzene in Fig. 1b is an appealing representation of its aromaticity, with a dark blue circle reminiscent of a Clar π-sextet.

A series of contorted PAHs for which structural parameters had been previously determined by single-crystal X-ray diffraction, electrostatic potential analyses were made available, or the delocalization of their electrons had been evaluated by other methods, were examined. The analyses of their 3D IMS contour maps showed systematic consistency with previous data, while providing an original point of view that allowed for new insights. More generally, these maps allowed an immediate and intuitive perception of the electron delocalization in the considered molecules. In just a glimpse, subtle variations can be discerned in the delocalization, from weak local π-bonding to aromatic-like circuits. It was recently identified that 2D IMS contour maps of some planar PAHs corroborate the predictions of Clar for their resonance structures.^44^ This pictorial feature is also present in the 3D IMS contour maps presented herein. However, the Clar π-sextet model^6,58^ was elaborated for planar PAHs and its extension to contorted molecules is not without some pitfalls.

The interactive 3D IMS maps of all molecules discussed in this article, and the procedure for viewing (and modifying) them, are available as ESI.† The reader is advised to take full advantage of this.

## Results and discussion

Pseudo-van der Waals surfaces composed of overlapping 1 Å radius spheres of *Bq* were generated using a purpose-made code and the computed IMS values were plotted using a neutral plus three shades of blue colour scale for the positive values, and a symmetric red colour scale for negative values when applicable. The small magnitude positive and negative values of IMS (|IMS| < 5.5 ppm) are shown with the same neutral colour in the maps presented herein. Of course, other fields than IMS can be visualized, for instance anisotropic NICS values (see section 4 in the ESI† for examples and comments), and the described pseudo-van der Waals method can be applied to any hydrocarbon independently of its geometry. To help at identifying the different recurring delocalization patterns discussed along the text, the IMS maps of representative simple alkenes and small planar PAHs were generated (Fig. S3 in the ESI†).

**Fig. 2:**
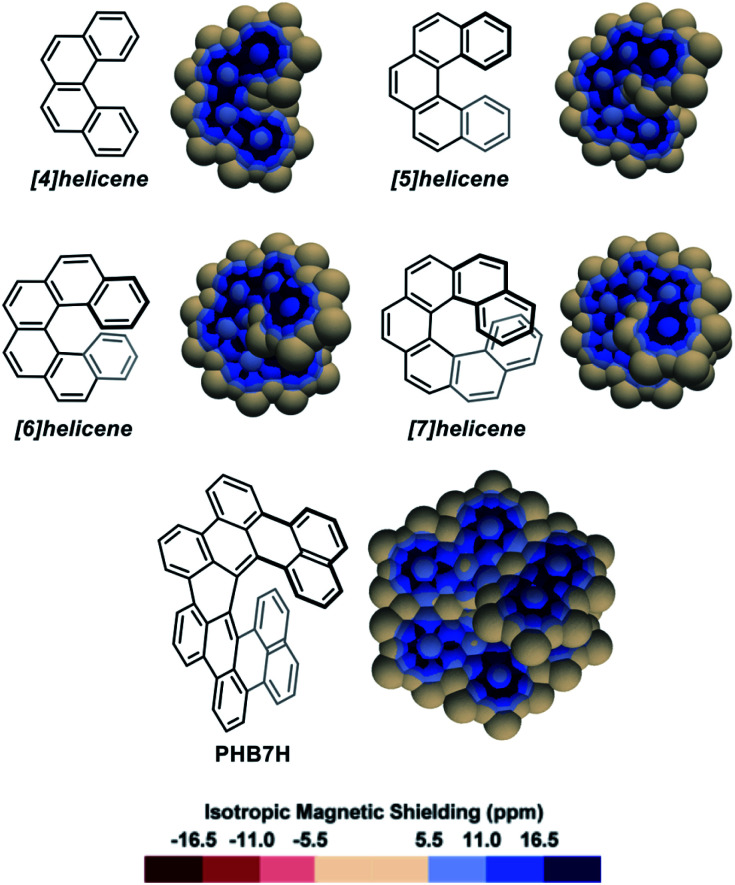
3D IMS contour maps of [*n*]helicenes and *peri*-hexabenzo[7]helicene (**PHB7H**) calculated at the B3LYP-GIAO/6-311++G**//B3LYP-D3BJ/def2-SVP level of theory.

As a first approach towards the analysis of contorted PAHs, simple [*n*]helicenes were examined.^51,57,59–62^ The 3D IMS maps of [4]-, [5]-, [6]-, and [7]helicene were generated and are reproduced in Fig. 2. The immediately perceptible feature of the maps is the unequal IMS values on the two sides of each individual ring (*C*_2_-symmetry), with more intense IMS values on the outer shell of the molecules, reflecting non-equivalent local magnetic environments above and below each ring. As the helix grows, ring stacking and the increasing effect of σ electrons (due to curvature) result in larger shielding cones. Consequently, anomalously large IMS values on terminal rings are obtained. This phenomenon has been studied previously.^57,60,62^ For instance, it is clearly visible in the 3D IMS contour map of [7]helicene, especially at the internal faces of the terminal rings. For comparative purpose, Kekulé, Clar, and ring bond order^63^ (RBO) analyses of [5]helicene are provided in the ESI (Fig. S4†). The delocalization patterns visualized in Fig. 2 for [*n*]helicenes match with the experimentally determined rate factor of all positions of [*n*]helicenes in some electrophilic aromatic substitution reactions,^64–66^ with the H-substituted carbon atoms showing the less delocalization over them in the maps being among the most reactive positions.

The IMS map of *peri*-hexabenzo[7]helicene (**PHB7H**),^67^ a laterally π-extended [7]helicene, reveals a different delocalization pattern at the [7]helicene edge induced by its π-extension. Separated phenanthrene-like and naphthalene-like π systems connected by carbon–carbon bonds having nearly no π character are now visible (as for perylene in Fig. S3 in the ESI†). This is a known feature for some classes of PAHs.^68,69^ Previous current densities analysis in **PHB7H** have allowed identifying weak paratropic currents at the inner rim of the central six-membered rings having no double bonds in the Lewis structure in Fig. 2.^67^ These currents induce negative IMS values of small magnitude not displayed in the IMS contour maps shown in Fig. 2 but visible using a modified colour scale (see Fig. S9 in the ESI†). This is a general feature of the maps presented herein, small effects are visualized with the neutral colour. However, the colour scale of all maps can be changed and adjusted at will, for instance to visualize weakly deshielded areas induced by small paratropic currents. See this and other selected examples in Section 5 in the ESI.† The example of **PHB7H** nicely illustrates the dramatic effect of π-extension in the delocalization patterns of molecules containing helical edges.

**Fig. 3:**
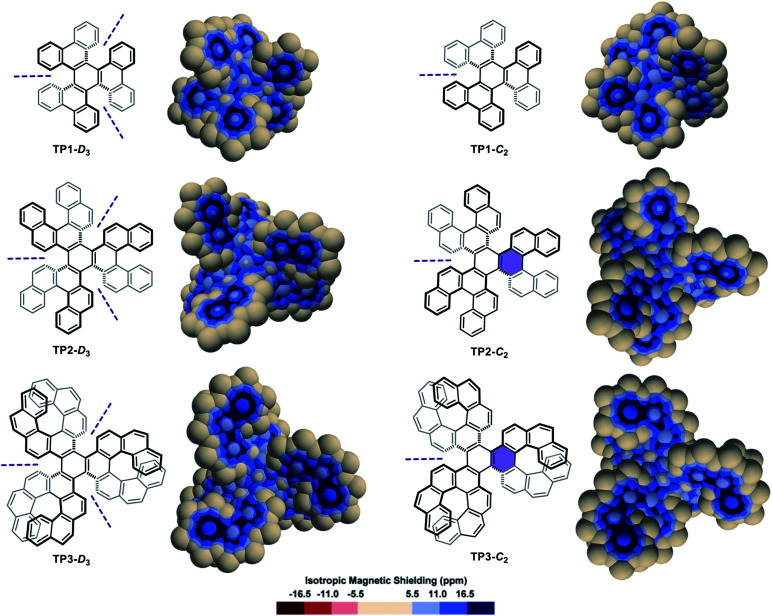
3D IMS contour maps of triphenylene-centred multi-helicenes, pairs of diastereomers, calculated at the B3LYP-GIAO/6-311++G**//B3LYP-D3BJ/def2-SVP level of theory. *C*_2_ symmetry axes are noted with purple dashed lines, and rings with remarkable torsion angles are highlighted with a purple background.

A series of diastereomeric triphenylene-based multi-helicenes with gradually increasing π-elongations and torsions were analysed (Fig. 3). The IMS maps of **TP1-D3** and **TP1-C2**,^70–72^ the two diastereomers of hexabenzotriphenylene, show benzene-like delocalization on the six rings at their periphery. Contrastingly, reduced delocalization is observed at the central rings that show three well-localized double bonds, confirming a marked 1,3,5-cyclohexatriene (the non-resonant Kekulé benzene) character of these rings (compare with *s-trans*-(*E*)-1,3,5-hexatriene in Fig. S3 in the ESI†). The least aromatic rings are the three rings surrounding the central one, which are incidentally the most twisted rings in the molecules. There are subtle but meaningful differences between the maps of **TP1-D3** and its diastereomer **TP1-C2**. Notably the central ring in **TP1-C2** shows comparatively more intense delocalization than the central ring in **TP1-D3**. The π-elongated analogues **TP2-D3**,^73,74^**TP2-C2**,^73^**TP3-D3**,^75,76^ and **TP3-C2** (ref. 76) show comparable patterns to those of their smaller analogues **TP1-D3** and **TP1-C2**. Notably, **TP2-C2** and **TP3-C2** embed the most distorted ‘benzene’ rings currently known, with torsion angles culminating at 35.7° and 36.9°, respectively (shown in purple colour in Fig. 3),^73,76^ which translate into weakened delocalization over these rings. Comparisons of the maps of **TP1-D3**, **TP2-D3**, and **TP3-D3**, and their *C*_2_-symmetric diastereomers, with those of phenanthrene (Fig. S3 in the ESI†), [5]helicene, and [7]helicene respectively, show that the global delocalization patterns in these multi-helicenes are approximately the three-fold version of the delocalization patterns of their parent molecules attached around a contorted 1,3,5-cyclohexatriene ring. For comparative purpose, the Clar, Kekulé, and RBO analyses of **TP1**, **TP2** and **TP3** are provided in the ESI (Fig. S6 and S7†).

**Fig. 4:**
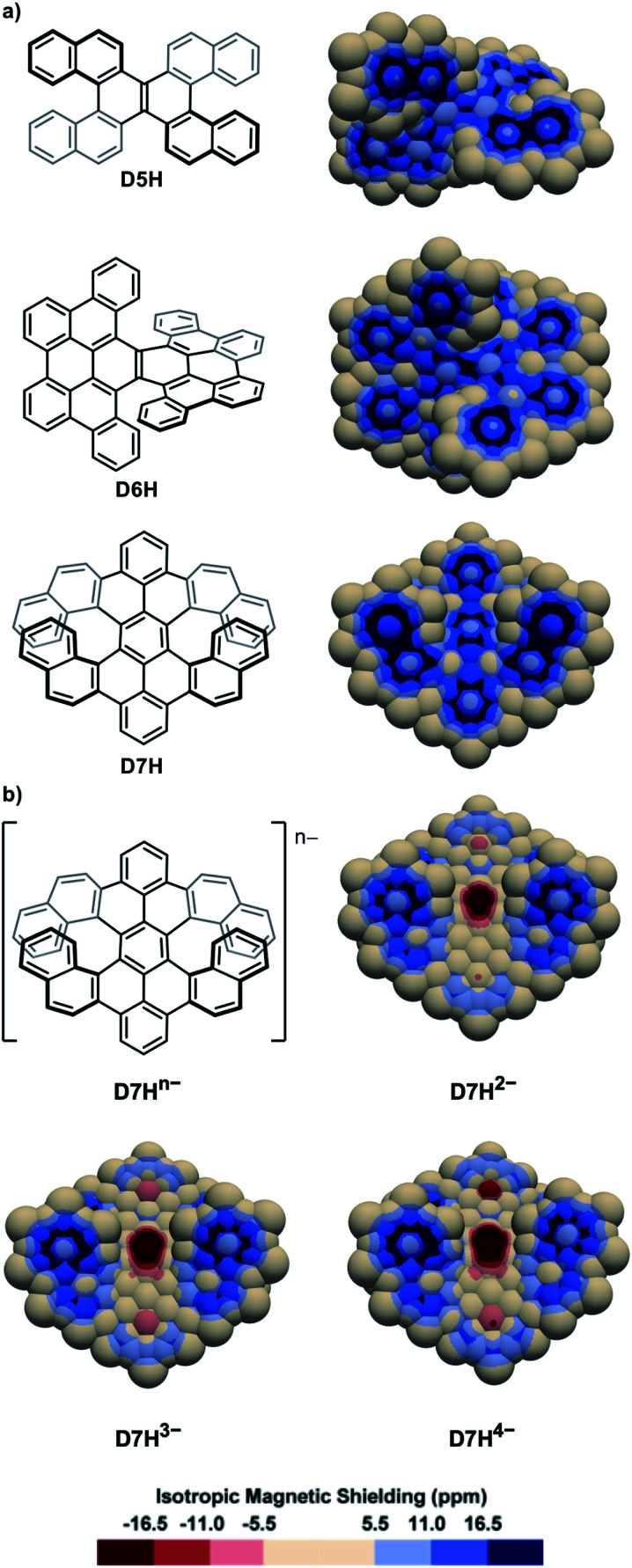
3D IMS contour maps of double helicenes and some corresponding anions calculated at the B3LYP-GIAO/6-311++G**//B3LYP-D3BJ/def2-SVP level of theory. (a) Neutral molecules. (b) Anionic analogues of **D7H**.

The IMS map of the double [5]helicene **D5H**^77^ (Fig. 4a) resembles that of its parent [5]helicene, with greater differences between the two faces of the peripheral rings, and a marked olefin-type character of the central bond. The map of the laterally π-extended double [6]helicene **D6H**^78^ (Fig. 4a) shows an organization of its π system with four phenanthrene-type domains attached to a moderately delocalized naphthalene core, and two carbon–carbon bonds at the external edges with very poor π character. The map of the π-extended double [7]helicene **D7H**^79^ is comparable showing separated π systems and some non-aromatic rings. The two-, three-, and four-fold reduction of **D7H** can produce the anions **D7H**^2−^, **D7H**^3−^, and **D7H**^4−^, respectively,^80^ the IMS maps of which are shown in Fig. 4b. These maps differ dramatically from that of the neutral molecule **D7H** with now large areas of intense negative IMS values (dark red colour) all over the central rings indicating the pronounced antiaromatic character of these rings. Negative IMS of lesser magnitude (red and light red colour) accounting for lower but significant antiaromatic character are also visible at the distal rings of the peropyrene core (top and bottom rings in Fig. 4b). The antiaromatic character of the central rings, and to some extent of the two distal rings of the peropyrene core, in anions **D7H**^2−^, **D7H**^3−^, and **D7H**^4−^ could not be perceived from their structural analyses or their electrostatic potential maps, whereas 3D IMS contour maps do this well. The electrostatic potential maps of **D7H** and its anions^80^ showed that the negative charges in the reduced species are likely distributed over the peropyrene cores, while their 3D IMS contour maps show well localized areas of intense negative IMS. Formally, this corresponds to an 8-electron Hückel antiaromatic central ring and poorly delocalized peropyrene core. This indicates that electrostatic potential maps not necessarily correlate with IMS maps. Both analytical methods are complementary to analyse electronic properties in contorted PAHs.

**Fig. 5:**
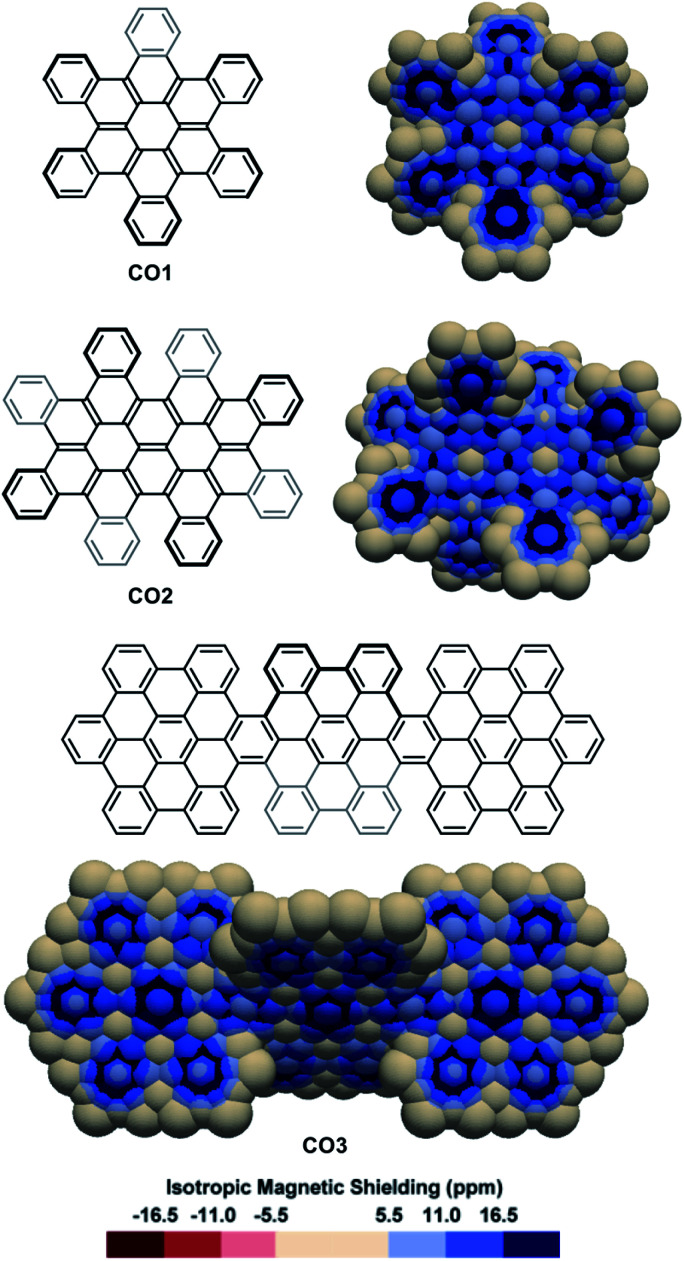
3D IMS contour maps of coronene-based nanographenes embedding helicenes at edges calculated at the B3LYP-GIAO/6-311++G**//B3LYP-D3BJ/def2-SVP level of theory.

The IMS maps of *cata*-hexabenzocoronene **CO1** and its two-fold analogue **CO2** were also analysed (Fig. 5, top).^81,82^ The Clar analysis of **CO1** was previously found to match well with its NICS analysis,^50,83^ and expectedly, the 3D IMS contour map of **CO1** was found in agreement with its Kekulé, RBO, and Clar analyses (Fig. S5 in the ESI†). In **CO1** and **CO2**, delocalization is visibly repulsed to the rings at the edges (with non-equivalent IMS of their faces), probably reflecting the existence of macrocyclic diatropic currents at the periphery of the molecule that can be perceived in the maps by a continuum of blue colour. This leaves non-aromatic rings at the centre of the coronene units visualized by large neutral colour areas over these rings. In alternative representations of the 3D IMS contour maps of **CO1** and **CO2** (Fig. S10 in the ESI†), small magnitude deshielded areas induced by low intensity paratropic currents can be visualized at the centre of these rings. In line with this, the bonds radiating from the central rings of the coronene units show discernible localized π character. The IMS map of the three-fold *peri*-hexabenzocoronene compound **CO3**^84^ (Fig. 5, bottom) shows that the central rings of the coronene units are now the most aromatic in the structure and that they are surrounded by six non-aromatic poorly resonating rings. Apart from the information on stereogenicity, the pictorial rendering of the 3D IMS contour map of **CO3** (a *fully benzenoid* PAH^58^) agrees well with its Clar structure. The differences between the delocalization patterns at the [5]helicene edges (fjord regions) in **CO2** and **CO3** and the delocalization in [5]helicene itself (Fig. 2) illustrate again the important effect of lateral π-extension in the electronics of contorted PAHs.

**Fig. 6:**
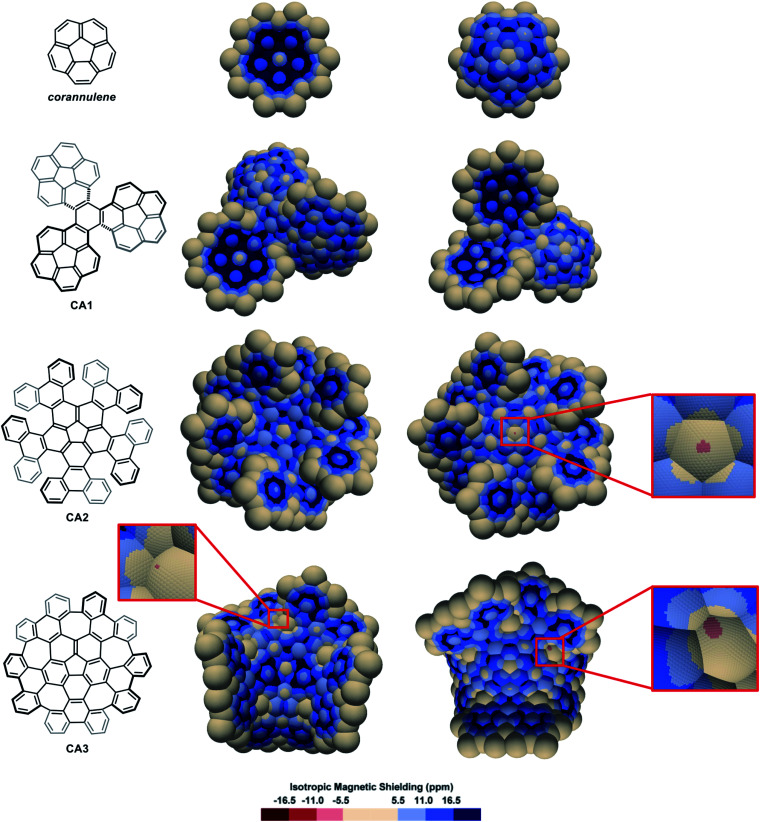
3D IMS contour maps of corannulene and corannulene-containing nanographenes calculated at the B3LYP-GIAO/6-311++G**//B3LYP-D3BJ/def2-SVP level of theory. Both faces of the molecules are shown (horizontal flip for the maps on the right).

The IMS map of corannulene (Fig. 6) shows large circular areas of intense shielding on the concave face indicating intense delocalization, and a more localized character of the π electrons on the convex face. This reflects the conical orientation of the π system in corannulene and the greater orbital overlaps on the concave face with more electron density. Corannulene cyclotrimer **CA1**^85^ (Fig. 6) shows comparable features with a more complex situation at the triphenylene core joining the three corannulene moieties. As for the multi-helicenes in Fig. 3, the central ring in **CA1** has some 1,3,5-cyclohexatriene character, now with *C*_1_-symmetry. In the corannulene-centred multi-helicene **CA2**^86^ (Fig. 6) the corannulene core shows significantly lower IMS and weaker delocalization than in corannulene itself and the corannulene units in **CA1**. Only localized resonance is visible on its concave face, and even a small area at the middle of its convex face with significantly negative IMS values (light red colour) pointing out for possibly significant paratropic current on this face of the ring. The graphitized version of multi-helicene **CA2** is nanographene **CA3**^87^ (Fig. 6) that contains both five-membered and seven-membered rings. The differences between the maps of **CA2** and **CA3** are essentially due to their different conformational or configurational arrangements and symmetry. Notably none of the seven-membered rings in **CA3** shows substantial delocalization as visualized by large neutral colour areas. However, some small light red colour areas are sharply localized over one face of two of the seven-membered rings indicating significant negative IMS at these locations. During the preparation of this manuscript, π-electron delocalization in **CA3** was investigated by complementary computational methods,^88^ showing full consistency with its map in Fig. 6 (see also Fig. S11 in the ESI†). Notably, an odd macrocyclic 75 π-electron aromatic circuit was identified by the authors in **CA3**, which is also perceptible in its IMS map, as visualised by a continuum of blue colour, with a greater distinction between contributions from each individual face of the molecule allowed by the 3D IMS contour map. Looking back at the map of **CA2** in Fig. 6, a quantitatively similar, different in shape and chiral too, macrocyclic 75-electron aromatic circuit is perceptible along the edge of the molecule.

**Fig. 7:**
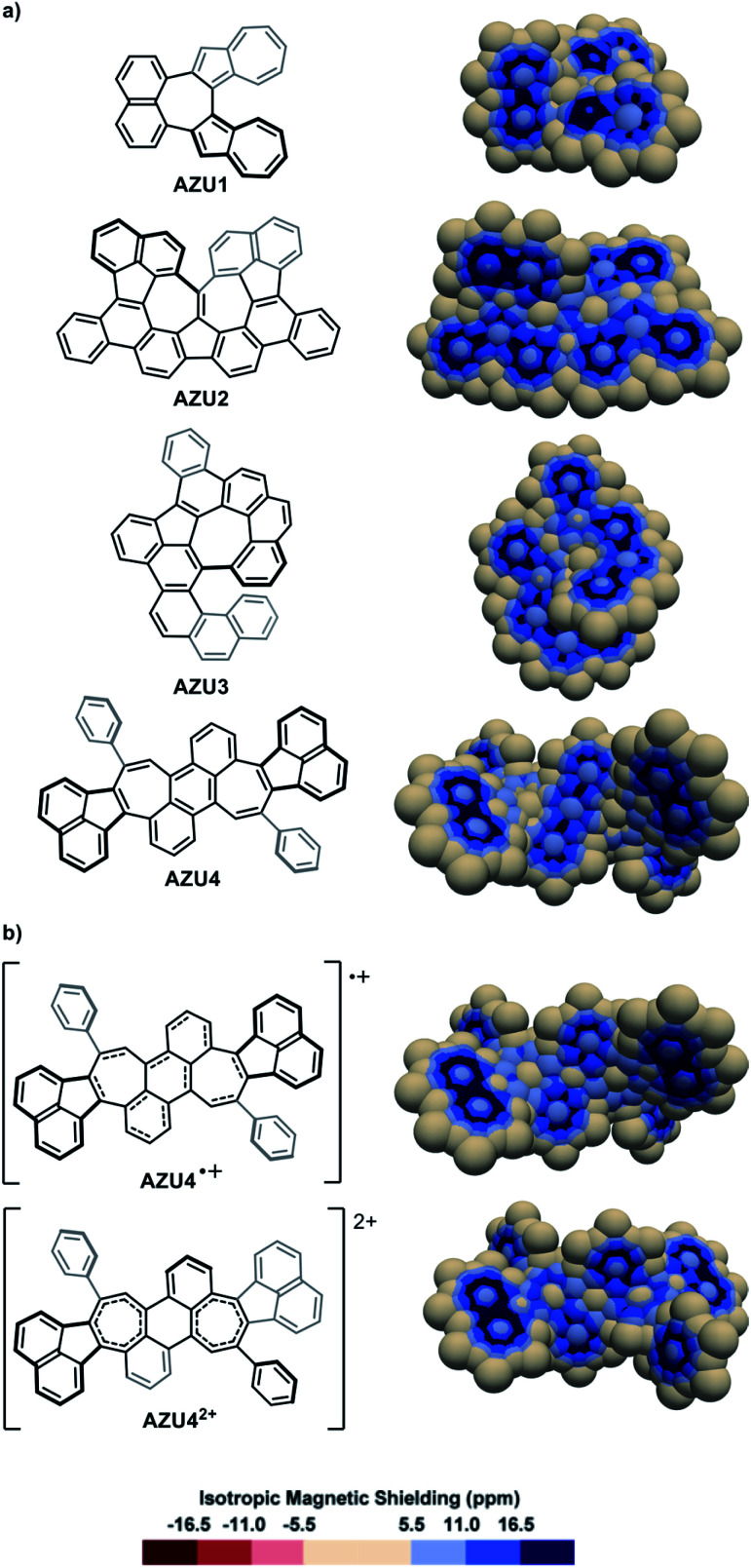
3D IMS contour maps of azulene-containing hydrocarbons and some corresponding cations calculated at the B3LYP-GIAO/6-311++G**//B3LYP-D3BJ/def2-SVP level of theory. (a) Neutral molecules. (b) Cationic analogues of **AZU4**.

The IMS maps of the azulene-containing molecules **AZU1**,^89^**AZU2**,^90^**AZU3**^91^ and **AZU4**^92^ are shown in Fig. 7a. **AZU1** shows three separated bicyclic aromatic delocalization systems: one naphthalene-like and two azulene-like (compare with naphthalene and azulene in Fig. S3 in the ESI†), with a non-aromatic central seven-membered ring. Contrastingly, for the three other molecules **AZU2**, **AZU3**, and **AZU4**, local aromaticity is nearly exclusively distributed over some of the six-membered rings in the structures, with the azulene moieties showing practically no aromaticity and poor π character of their carbon–carbon bonds. It is remarkable how the IMS contour maps of these molecules reflect their “azulene-containing” or not character from the delocalization point of view (see also Fig. S12 in the ESI†). The one- and two-electron oxidations of **AZU4** produced the cationic molecules **AZU4**^+^ and **AZU4**^2+^.^92^ The IMS maps of these cationic molecules are represented in Fig. 7b. An important consequence of the one-electron oxidation is the reorganization of delocalization in **AZU4**^+^ as compared to **AZU4** with the central benzene ring losing some aromatic character to the benefit of the two vicinal seven-membered rings. This modification in the distribution of local aromaticity is further accentuated in the two-electron oxidized dication **AZU4**^2+^, forced to adopt another conformation. Its IMS map shows a non-aromatic central benzene ring and two significantly shielded seven-membered rings showing some aromatic character.

## Conclusions

The analysis of electron delocalization in a selection of contorted PAHs was performed through visualization of high-resolution 3D IMS contour maps built around the molecules using a pseudo van der Waals approach. These maps have a pictorial rendering reminiscent of the Clar π-sextet model, facilitating an immediate interpretation. The selected molecules are often large and chiral, possibly embedding extreme torsion and/or charges, and were otherwise fully characterized. Their maps showed intuitive and information-rich representations of their local and global electron delocalization circuits and aromatic character, in full agreement with their previously determined properties, while providing an original and largely complementary perspective. Important effects of π-extension and/or redox processes in contorted PAHs could be visualized, so could be the subtle differences in electron delocalization between diastereomers of some chiral multi-helicenes. The 3D IMS contour maps stress out the necessity to look at the problem in 3D when evaluating electron delocalization in contorted PAHs. This approach may reveal valuable for the analysis of other contorted π systems.

## Data availability

The optimized geometries (geometries.xyz) and the IMS data (vtk_files.zip) of all molecules are provided with the ESI.†

## Author contributions

A. A. performed the calculations. D. H.-R. analyzed the physical concepts and mathematical formalism. Y. Ca wrote the purpose-made code. Y. Co conceived the research hypothesis. All authors contributed to the development of the work and co-wrote the manuscript.

## Conflicts of interest

There are no conflicts to declare.

## Supplementary Material

SC-012-D1SC03368A-s001

SC-012-D1SC03368A-s002

SC-012-D1SC03368A-s003

SC-012-D1SC03368A-s004

SC-012-D1SC03368A-s005

SC-012-D1SC03368A-s006

SC-012-D1SC03368A-s007

SC-012-D1SC03368A-s008

SC-012-D1SC03368A-s009

SC-012-D1SC03368A-s010

SC-012-D1SC03368A-s011

SC-012-D1SC03368A-s012

SC-012-D1SC03368A-s013

SC-012-D1SC03368A-s014

SC-012-D1SC03368A-s015

SC-012-D1SC03368A-s016

SC-012-D1SC03368A-s017

SC-012-D1SC03368A-s018

SC-012-D1SC03368A-s019

SC-012-D1SC03368A-s020

SC-012-D1SC03368A-s021

SC-012-D1SC03368A-s022

SC-012-D1SC03368A-s023

SC-012-D1SC03368A-s024

SC-012-D1SC03368A-s025

SC-012-D1SC03368A-s026

SC-012-D1SC03368A-s027

SC-012-D1SC03368A-s028

SC-012-D1SC03368A-s029

SC-012-D1SC03368A-s030

SC-012-D1SC03368A-s031

SC-012-D1SC03368A-s032

SC-012-D1SC03368A-s033

SC-012-D1SC03368A-s034

SC-012-D1SC03368A-s035

SC-012-D1SC03368A-s036

SC-012-D1SC03368A-s037

SC-012-D1SC03368A-s038

SC-012-D1SC03368A-s039

SC-012-D1SC03368A-s040

SC-012-D1SC03368A-s041

SC-012-D1SC03368A-s042

SC-012-D1SC03368A-s043

SC-012-D1SC03368A-s044

SC-012-D1SC03368A-s045

SC-012-D1SC03368A-s046

SC-012-D1SC03368A-s047

SC-012-D1SC03368A-s048
