# Supplementary material for: Visualizing electron delocalization in contorted polycyclic aromatic hydrocarbons
Source: Chem Sci. 2021 Sep 8;12(39):13092–100. doi: 10.1039/d1sc03368a (PMC8513938; doi:10.1039/d1sc03368a)
Supplement: SC-012-D1SC03368A-s002 [file SC-012-D1SC03368A-s002.pdf]

Electronic Supplementary Material (ESI) for Chemical Science.
This journal is © The Royal Society of Chemistry 2021

ELECTRONIC SUPPLEMENTARY INFORMATION

Visualizing electron delocalization in contorted polycyclic aromatic hydrocarbons

Albert Artigas, Denis Hagebaum-Reignier, Yannick Carissan* and Yoann Coquerel*

Aix Marseille Université, CNRS, Centrale Marseille, ISM2, 13397 Marseille, France

yannick.carissan@univ-amu.fr
yoann.coquerel@univ-amu.fr

Table of Content

1. Computational details S2
2. Maps of simple alkenes and small planar PAHs S5
3. Kekulé, Clar and ring bond order (RBO) analyses of selected molecules S6
4. Examples of 3D anisotropic NICS contour maps S10
5. 3D IMS contour maps of selected contorted hydrocarbons using an alternative scale S12
6. Visualization procedure for .vtk files using Paraview S14
7. References S18

1. Computational details

All geometry optimizations were performed with the Gaussian 16 package[1] by using the B3LYP hybrid density functional[2,3] with the D3BJ correction for dispersion[4] and the def2-SVP basis set[5] without symmetry constraints. The corresponding .xyz files are provided as electronic supplementary information. Analytical Hessians were computed to confirm that the optimized structures are indeed minima (zero imaginary frequencies). When necessary, the chemical structure of the molecules studied was slightly modified (i.e., removal of alkyl substituents) prior to optimization to keep the polycyclic network only. Some dummy nuclei were empirically added at selected barycentres (Fig. S1) to produce smoother surfaces and get rid of misleading (de)shielding information at the edges arising from σ C–C and C–H bonds. Pseudo-van der Waals surfaces of *Bq* made of overlapping spheres of 1 Å radius centred on the nuclei were generated using a purpose-built code. For the effect of the radii of the spheres see Fig. S2. NMR-GIAO[6] calculations at every *Bq* were performed at the B3LYP-GIAO/6-311++G(d,p)[7] level and using the CPHF(Separate) keyword to improve accuracy. NMR-GIAO Gaussian 16 input files were split to limit the number of *Bq* in each calculation at max. 30.000 *Bq*/input; the number of *Bq* computed for each map is shown in Table S1. All IMS data obtained from NMR-GIAO Gaussian 16 calculations were collected in vtk-formatted files using a purpose-made code. Paraview visualization software was used to visualize and prepare all pictures presented in this work. See the visualization tutorial in section 6 of this document.

Rings: Insert one dummy atom at the barycentre of 6 atoms, at the middle of each ring.

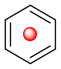

Zig-zag regions: Insert one dummy atom at the barycentre of 5 atoms.

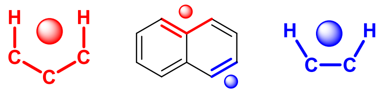

M or K regions: Insert one dummy atom at the barycentre of 4 atoms.

Bay regions: Insert 1 dummy atom at the barycentre of 6 atoms.

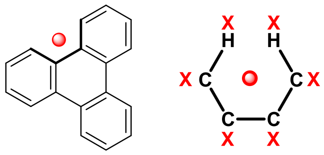

Fjörd regions: Insert 1 dummy atom at the barycentre of 4 atoms (green) and 2 dummy atoms at the barycentre of 2 groups of 5 atoms (red and blue).

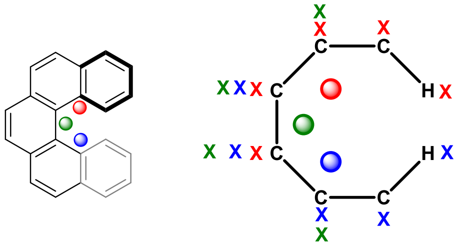

Inner rim of [6]helicene moieties: Insert 1 dummy atom at the barycentre of 3 groups of 5 atoms.

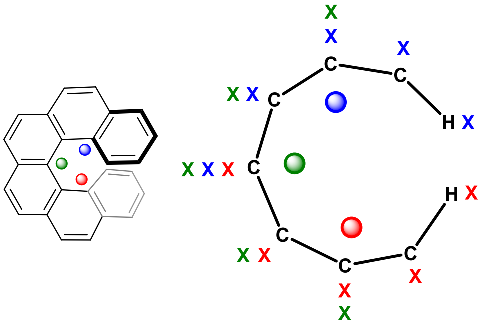

Inner rim of [7]helicene moieties: Insert 1 dummy atom at the barycentre of 6 atoms (green) and 2 dummy atoms at the barycenter of 2 groups of 5 atoms (red and blue).

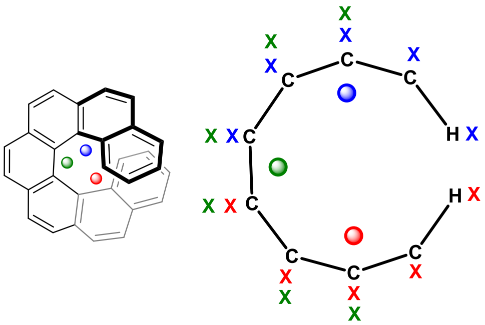

Fig. S1. Criteria applied to add the dummy nuclei. The Chemcraft visualization software[8] was used to compute the cartesian coordinates of each selected barycentre. Only six-membered rings containing examples are shown; analogous criteria were used to compute the position of the dummy nuclei in molecules containing five- and seven-membered rings.

During the optimization phase of this work, several options were evaluated for the radius of the spheres, from 0.90 Å to 1.15 Å (Fig. S2), showing that a 1 Å radius gives the best rendering.

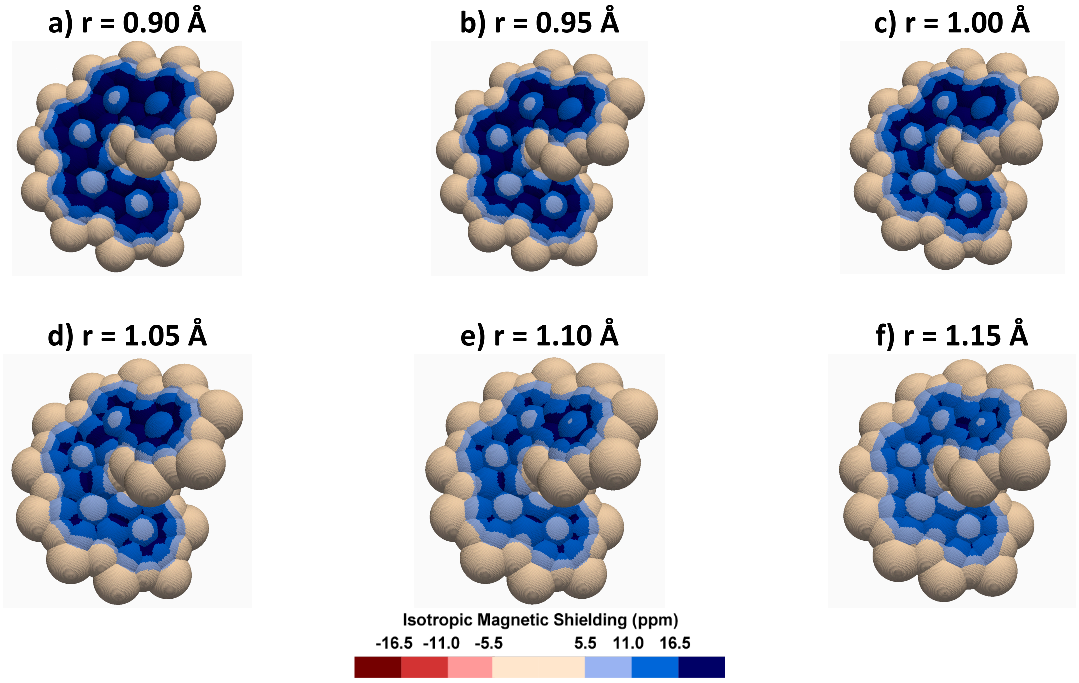

Fig. S2. B3LYP-GIAO/6-311++G(d,p)//B3LYP-D3BJ/def2-SVP computed 3D IMS contour maps of [5]helicene: effect of the pseudo van der Waals spheres radius (r).

The number of *Bq* computed for each map (Table 1) it is about two orders of magnitude smaller than the number of *Bq* that would be required to plot IMS isovalue surfaces (ICSS) with comparable resolution.

Table S1. Number of *Bq* computed for generating the IMS maps.

**[4]helicene**	70 340	**$D7H^{3-}$**	203 669
**[5]helicene**	84 835	**$D7H^{4-}$**	206 047
**[6]helicene**	99 504	**CO1**	164 541
**[7]helicene**	114 085	**CO2**	232 797
**PHB7H**	165 494	**CO3**	347 798
**TP1-D_3**	155 019	**corannulene**	71 248
**TP1-C_2**	153 621	**CA1**	194 859
**TP2-D_3**	238 740	**CA2**	278 296
**TP2-C_2**	237 882	**CA3**	252 352
**TP3-D_3**	321 701	**AZU1**	117 496
**TP3-C_2**	322 518	**AZU2**	166 511
**D5H**	155 161	**AZU3**	152 844
**D6H**	196 256	**AZU4**	199 659
**D7H**	202 835	**$AZU4^{+}$**	199 413
**$D7H^{2-}$**	203 319	**$AZU4^{2+}$**	198 630

2. Maps of simple alkenes and small planar PAHs

The 3D IMS contour maps of simple alkenes including ethylene, *s-trans*-1,3-butadiene, *s-cis*-1,3-butadiene, *s-trans*-(*E*)-1,3,5-hexatriene, and *s-trans*-(*Z*)-1,3,5-hexatriene were obtained (Fig. S3a). The maps of representative planar monocyclic and small polycyclic aromatic hydrocarbons including the cyclopentadienyl anion, the tropylium cation, naphthalene, azulene, phenanthrene, pyrene, triphenylene, perylene, and coronene were also generated (Fig. S3b). Overall, these maps reflect the electron delocalization patterns as expected. The corresponding .vtk files are also available in the ESI for 3D viewing and personalisation, see the visualization tutorial in section 6 of this document.

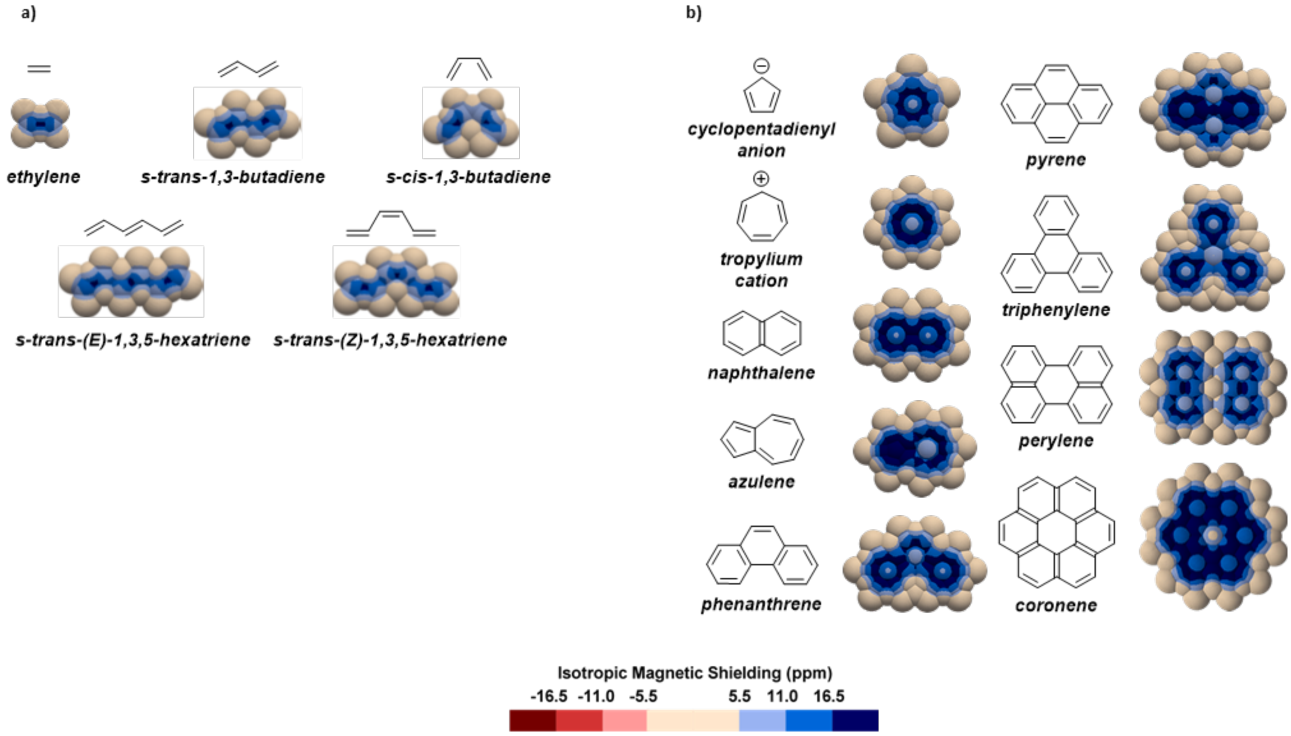

Fig. S3. 3D IMS contour maps of planar systems calculated at the B3LYP-GIAO/6-311++G**//B3LYP-D3BJ/def2-SVP level of theory. a) Representative alkenes. b) Representative monocyclic and small polycyclic aromatic hydrocarbons.

3. Kekulé, Clar and ring bond order (RBO) analyses of selected molecules

The Kekulé and ring bond order (RBO)[9] analyses of selected molecules are provided below (left column). The colour code is as follows:

- the green colour number at the top left corner is the total number of Kékulé structures, noted K, for the considered molecule.
- the green colour number at the centre of each ring is the number of times that a ring has 3 double bonds in it (a Clar π-sextet) as a percentage of K.
- the orange colour numbers over bonds are the number of times that a bond is a double bond as a percentage of K.
- the yellow colour number at the centre of each ring is the RBO of this ring; it is obtained by summing the six orange colour numbers over bonds for this ring and dividing this sum by 100.

The Clar analysis of the selected molecules are also provided below (right column). "Clar's rule states that the Kekulé resonance structure with the largest number of disjoint aromatic π-sextets, i.e., benzene-like moieties, is the most important for characterization of properties of polycyclic aromatic hydrocarbons (PAHs)."[10] The Clar structures shown below correspond to this definition.

An important feature of the Kekulé, RBO and Clar analyses is, as topological methods, they only account for connectivity. Geometry is not considered in these analyses.

[5]helicene

The Kekulé, RBO, and Clar analyses for [5]helicene are shown below (Fig. S4). Note that these analyses for [5]helicene and picene provide identical outcomes. While the Clar structure of [5]helicene is somehow extreme for the representation of its aromaticity, the Kekulé and RBO analyses provide a reasonable picture of it, comparable with the global picture conveyed by its 3D IMS contour map. The chirality of the π system of [5]helicene is only visible in the 3D IMS contour map though.

Kekulé and RBO analyses of [5]helicene:

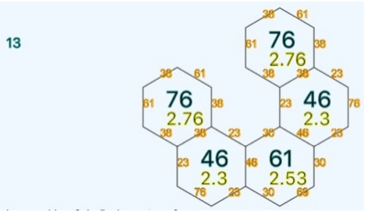

Clar analysis of [5]helicene:

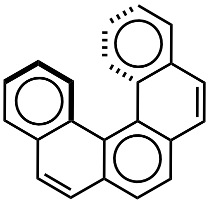

Kekulé and RBO analyses of picene:

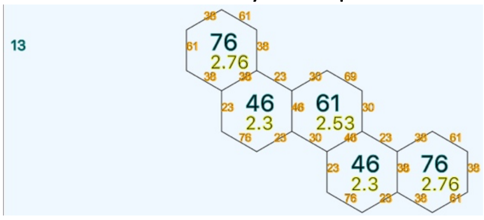

Clar analysis of picene:

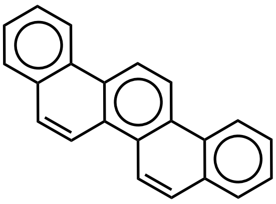

Fig. S4. Kekulé, RBO, and Clar analyses for [5]helicene.

CO1

The Kekulé, RBO, and Clar analyses for **CO1** are shown below (Fig. S5). It was previously shown that the Clar and NICS analyses of **CO1** agree well.[11,12] The global information conveyed by the 3D IMS contour map of **CO1** is in nice agreement with its Kekulé, RBO, and Clar analyses. The stereogenicity of the π system is only visible in the 3D IMS contour map though.

Kekulé and RBO analyses of **CO1:**

Clar analysis of **CO1:**[11,12]

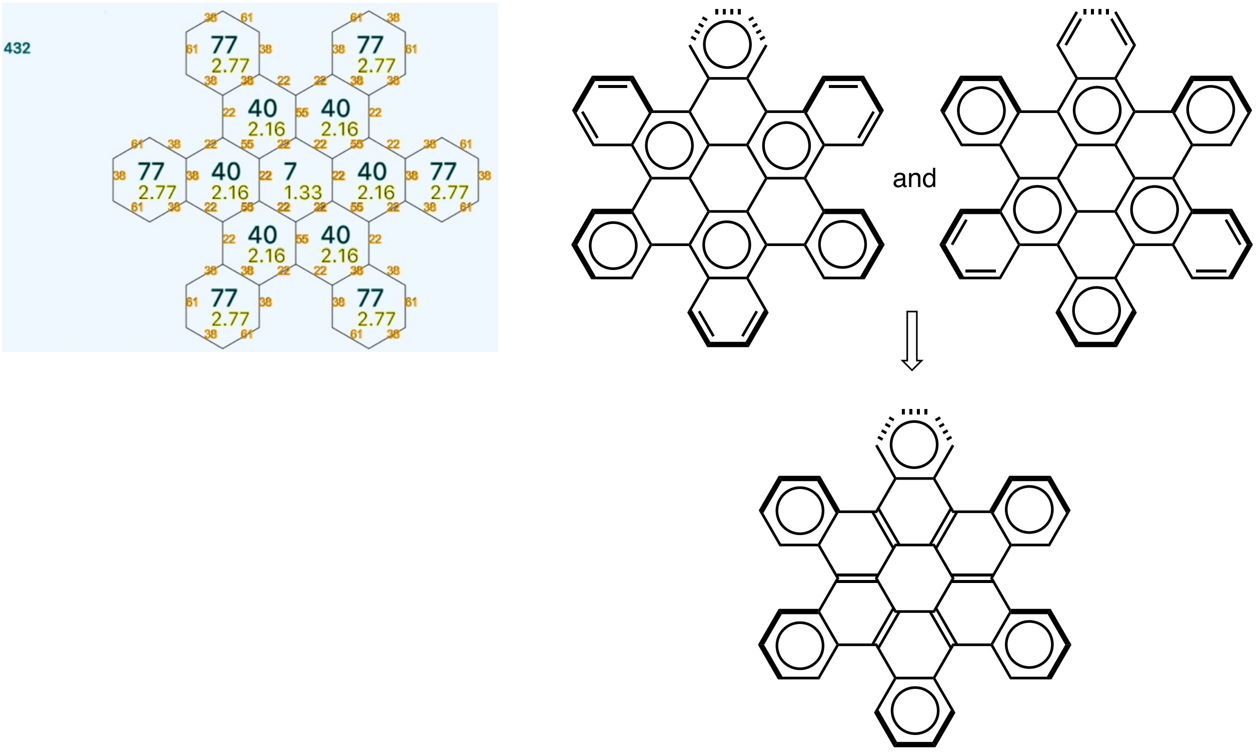

Fig. S5. Kekulé, RBO, and Clar analyses for **CO1**.

TP1, TP2, and TP3

The Kekulé, RBO, and Clar analyses for **TP1**, **TP2**, and **TP3** are shown below (Fig. S6). Note that these analyses are identical for the D_3- and C_2-symmetric diastereomers, and that the Kekulé and RBO analyses for **TP3** have been performed on a regioisomer with similar connectivity.

Kekulé and RBO analyses of **TP1**:

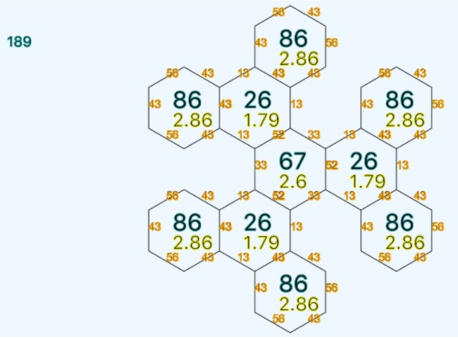

Clar analysis of **TP1** (D_3-symmetric diastereomer shown):

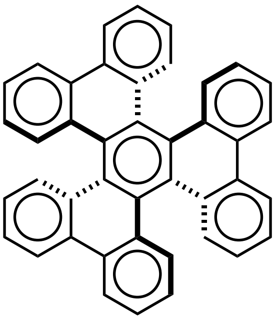

Kekulé and RBO analyses of **TP2:**

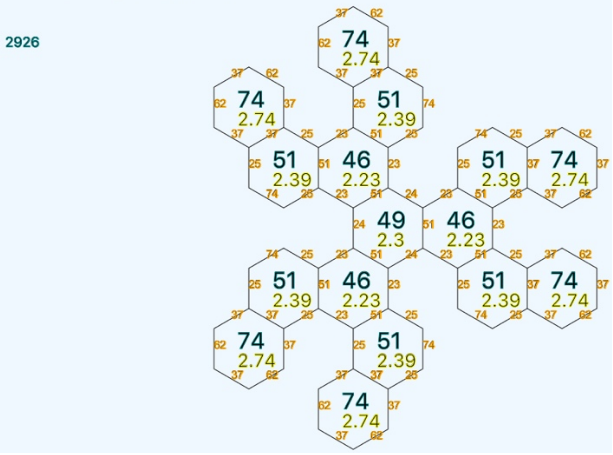

Clar analysis of **TP2** (D_3-symmetric diastereomer shown):

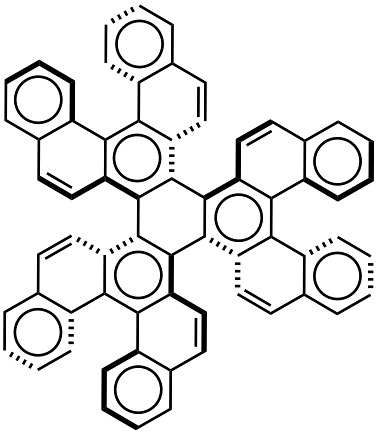

Kekulé and RBO analyses of **TP3:**

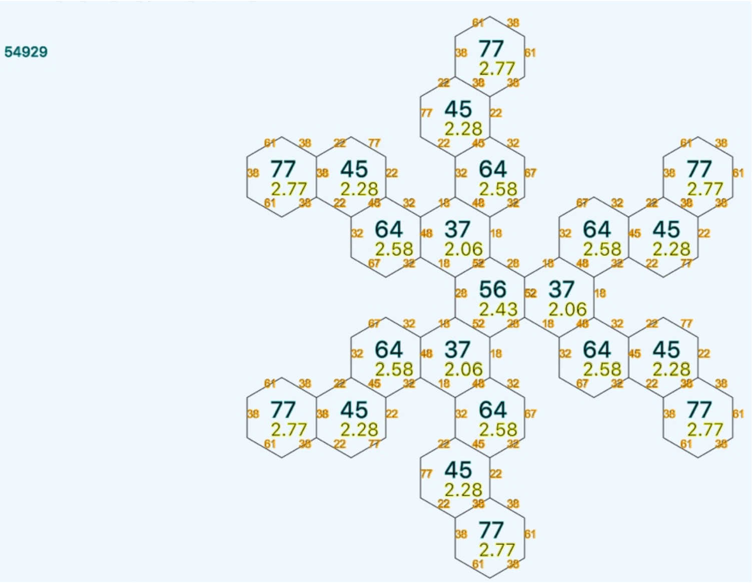

Clar analysis of **TP3** (D_3-symmetric diastereomer shown):

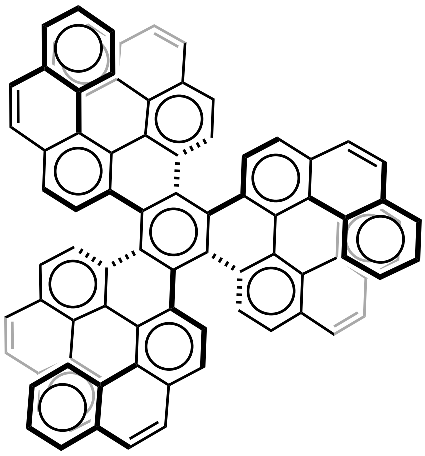

Fig. S6. Kekulé, RBO, and Clar analyses for **TP1**, **TP2**, and **TP3**.

The Kekulé, RBO, and Clar analyses of **TP1** agree relatively well with the global picture of aromaticity obtained from the 3D IMS contour maps of both its diastereomers, when not considering the information on chirality. Note that **TP1** is a so-called *fully benzenoid* PAH, meaning its Clar structure can be drawn without showing an isolated double bond and helping at identifying the most relevant Kekulé structure. **CO3** (Fig. 5 in the main text) is another example of *fully benzenoid* PAH.

The Kekulé, RBO, and Clar analyses of **TP2** globally failed at producing a reasonable pattern of its aromaticity, while the 3D IMS contour maps of **TP2-*D*$_3$** and **TP2-*C*$_2$** are incomparably informative on that matter. In the Kekulé and RBO analyses of **TP2**, the '**TP1** core' (**TP2** is a hexabenzo-**TP1**) is shown with nearly identical aromaticity over all rings, with Kekulé values ranging from 46 to 51, and RBO values ranging from 2.23 to 2.39. In sharp contrast, the information conveyed by the IMS maps show marked differences of local aromaticity of the rings. The Clar structure of **TP2** shown above with nine π-sextets is also misleading, localizing some π-sextets at the three rings surrounding the central one, while structural analyses of the diastereomers of **TP2**[13,14] show extreme torsion in these rings and their IMS maps show a non-aromatic character for these rings (which was confirmed by ACID analyses[15]). Looking obliquely at the Clar representation of **TP2**, we propose it is as depicted below (Fig. S7) instead of as shown above (Fig. S6), with now seven total π-sextets including six migrating sextets.

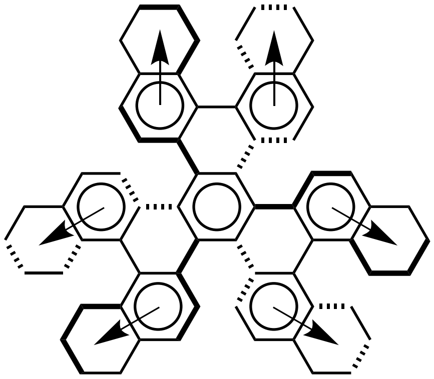

Fig. S7. Alternative Clar analysis of **TP2** (D_3-symmetric diastereomer shown)

The Kekulé, RBO, and Clar analyses of **TP3** show a better match with their corresponding 3D IMS contour maps as compared with **TP2**. The central rings of each [7]helicene moiety are the least aromatic ones according to all four models. All rings showing a Clar π-sextet have the highest RBO and %K values and show intense positive IMS values at some extent. It should be noted, however, that pronounced distortion of the central ring dramatically reduces its aromatic character. This feature is nicely perceptible in its 3D IMS contour map.

Altogether, this brief comparative study illustrates the limits of topological approaches for the analysis of contorted π systems.

4. Examples of 3D anisotropic NICS contour maps

Using the described pseudo van der Waals 3D mapping approach and a slightly modified procedure for the data collection, other fields than IMS (or $-NICS_{iso}$) can be visualized, for instance anisotropic $NICS_{zz}$ values. Representing $NICS_{zz}$ requires the definition of a z axis perpendicular to the π system along which the computational analysis is performed, which is not without causing problems for contorted systems, especially the severely contorted molecules discussed in the manuscript. The benefits of an isotropic/anisotropic description of the magnetic response in contorted molecules are a matter of debate, and there is not a single answer to the question of aromaticity in contorted PAHs. The principal matter of debate is what the best approximation is with the available tools and knowledge. With an isotropic approach, one does an approximation using averaged values along the three directions of space [IMS = σ_{iso} = $(\sigma_{xx} + \sigma_{yy} + \sigma_{zz}) / 3$]. With an anisotropic approach such as $NICS_{zz}$, one does the approximation of an arbitrarily chosen z axis for the analysis [$NICS_{zz} = -\sigma_{zz}$] while most electronic delocalization is occurring in nonplanar circuits not perpendicular to this axis and sometimes far from it.
To evaluate the anisotropic option, [5]helicene and **CA3** were selected as illustrative examples of moderately contorted and extremely contorted molecules, respectively. All sorts of data on the analysis of electron delocalization in these molecules are available for comparison (see references 51, 57, 59–62, and 88 in the main text). Their 3D $-NICS_{zz}$ contour maps were generated and are represented in Fig. S8.

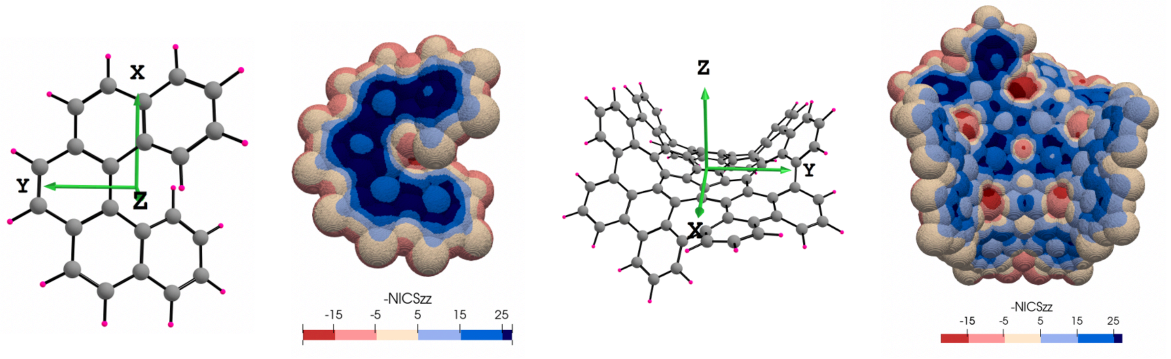

3D representation of $-NICS_{zz}$ on a surface around [5]helicene (1 Å sphere radius).

3D representation of $-NICS_{zz}$ on a surface around **CA3** (1 Å sphere radius).

Fig. S8. 3D $-NICS_{zz}$ contour maps of [5]helicene (left) and **CA3** (right).

Comparison with the 3D IMS contour maps of [5]helicene and **CA3** (Fig. 2 and 6 in the main text, respectively) illustrates the different outcomes obtained with the two approaches. Notably, some misleading information is visible in colour at the edges in the 3D $-NICS_{zz}$ contour maps that is not relevant to π electrons delocalization (contributions from σ electrons).
In [5]helicene, both $-NICS_{zz}$ and IMS contour maps are informative and convey reasonably reliable information. For generation of the –NICSzz contour map, a z axis perpendicular to the mean plane defined by the six carbon atoms of the central ring was arbitrarily selected. As a result, the two mean planes defined by the two terminal rings show an angle of ca. ±23° from perpendicularity to the z axis, inducing significant inaccuracies in the description of the electron delocalization in these rings.
In **CA3**, a suitable z axis may be chosen perpendicular to the mean plane defined by the central five-membered ring. Because **CA3** is very contorted, the mean planes defined by some of the peripheral rings show an angle < 40° (down to 35.9°) with the z axis, that is up to 54.1° away from perpendicularity to the z axis. These angle measurements were derived from the single crystal structure of **CA3** obtained by X-ray diffraction analysis (CCDC #919707). In other words, the electronic circuits responsible for the magnetic shielding/deshielding in the peripheral rings would be better analysed along the x or y axes depending on cases. Therefore, the $-NICS_{zz}$ map of **CA3** shows important variations of the magnetic shielding/deshielding that significantly reflect the local deviations from perpendicularity to the z axis and thus substantial contributions from σ electrons. The most remarkable differences between the $-NICS_{zz}$ (Fig. S8) and IMS (Fig. 6 in the main text) versions of the 3D contour maps of **CA3** are as follow:

- Some deshielded areas are visualized as light-to-dark red colour areas at the inner rim of the seven-membered rings in the 3D $-NICS_{zz}$ contour map of **CA3** while these are shown with a neutral colour meaning $|IMS| < 5.5$ in the corresponding IMS contour map. These deshielded areas exist in **CA3** and are possibly over-expressed in the $-NICS_{zz}$ map as analysed previously (see ref. 88 in the main text). Note that the deshielded areas are well accounted for in the 3D IMS contour map using a modified colour scale (see section 5 and Fig. S11).
- The unusual 75 π-electron aromatic circuit that was recently identified in **CA3** (see ref. 88 in the main text) cannot be visualized in the 3D $-NICS_{zz}$ contour map, while both faces of the magnetic shielding resulting from this delocalization circuit are nicely visible in the corresponding IMS map.
- In the 3D $-NICS_{zz}$ contour map of **CA3**, the subtle information induced by the chirality of the π system visible on the corresponding 3D IMS contour map is entirely masked by the important variations induced by the anisotropy of the analysis.

For the 3D visualization of electron delocalization through a magnetic shielding/deshielding analysis in contorted PAHs, we have come to favour the isotropic approach as the most viable option. This is especially true for some of the extremely contorted molecules discussed in the main text.

5. 3D IMS contour maps of selected contorted hydrocarbons using an alternative scale

In the 3D IMS contour maps presented in the main text, a neutral colour is employed to visualize small-magnitude IMS values (|IMS| < 5.5 ppm). In some cases, this may result in not displaying some relevant information, especially the deshielded areas induced by low-intensity paratropic currents that are often occurring at the inner rim of five-membered and seven-membered rings, and sparingly at the inner rim of six-membered rings, in neutral PAHs. In the pursuit of a detailed analysis, the colour scale of the 3D IMS contour maps can be easily modified to suppress the neutral colour and show the small-magnitude IMS values in light blue/red colour. In these versions of the 3D IMS contour maps, the weakly deshielded areas at the centre of some rings are clearly visualized (Fig. S9–S12). However, some misleading information not relevant to electron delocalization in the conjugated π system (but to σ electrons) also become visible at all edges of the molecules in these versions of the maps.

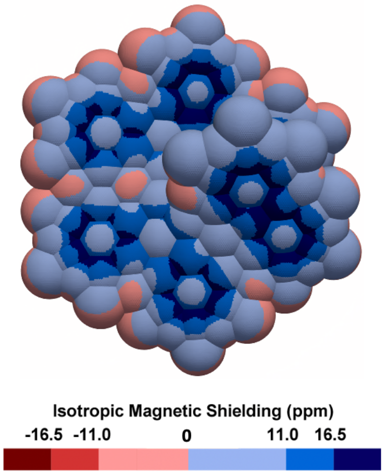

Fig. S9. 3D IMS contour map of **PHB7H** calculated at the B3LYP-GIAO/6-311++G**//B3LYP-D3BJ/def2-SVP level of theory.

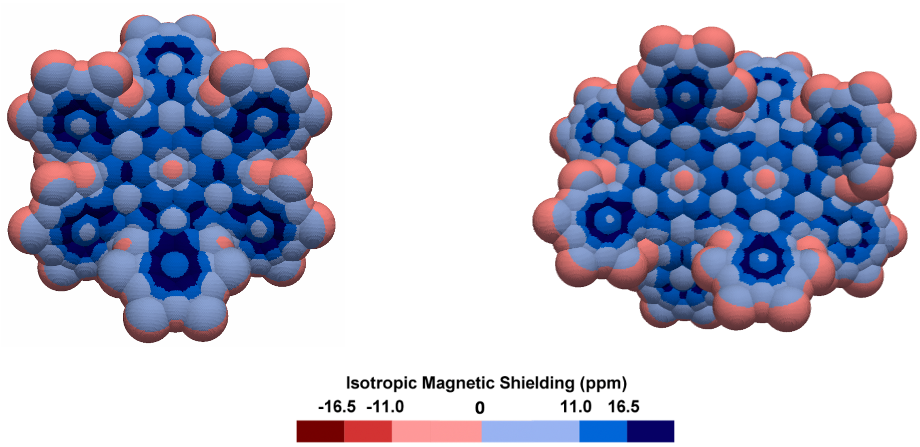

Fig. S10. 3D IMS contour maps of **CO1** (left) and **CO2** (right) calculated at the B3LYP-GIAO/6-311++G**//B3LYP-D3BJ/def2-SVP level of theory.

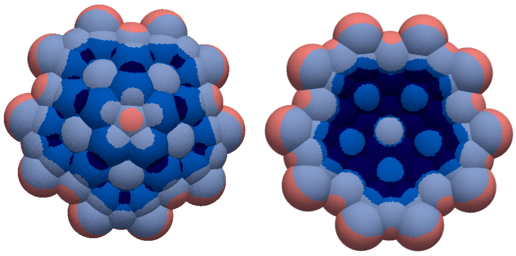

corannulene (both faces)

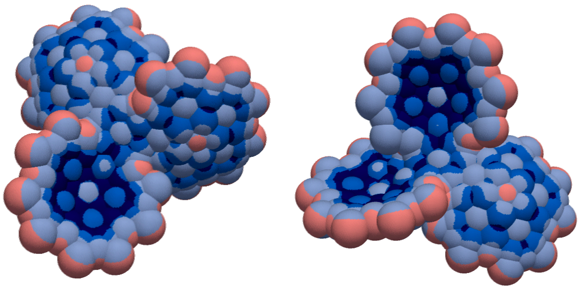

CA1 (both faces)

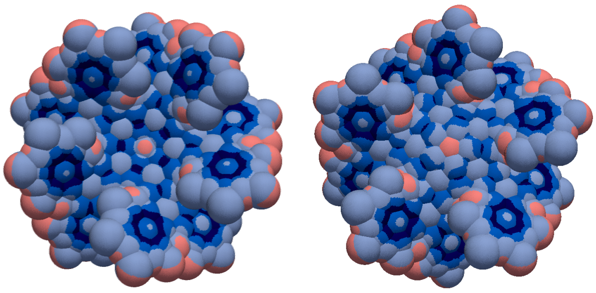

CA2 (both faces)

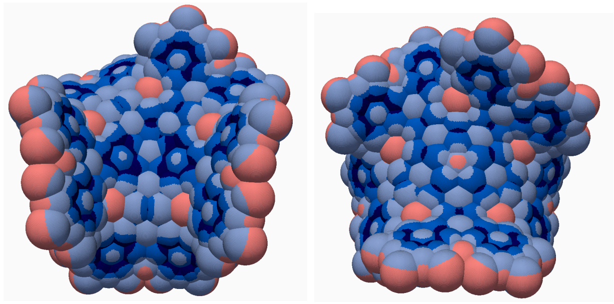

CA3 (both faces)

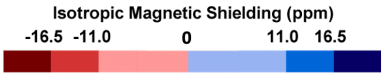

Fig. S11. 3D IMS contour maps of corannulene, **CA1**, **CA2**, and **CA3** calculated at the B3LYP-GIAO/6-311++G**//B3LYP-D3BJ/def2-SVP level of theory.

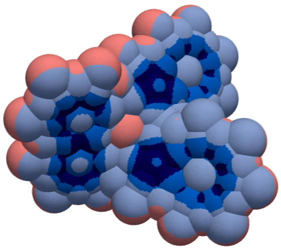

AZU1

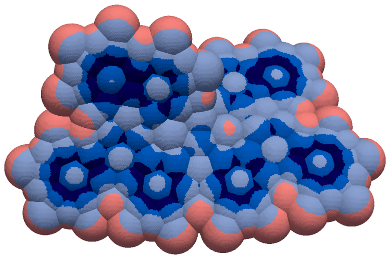

AZU2

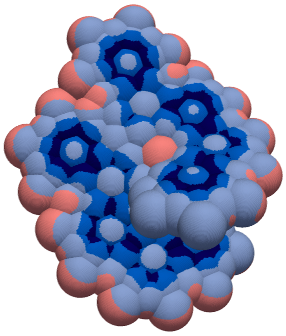

AZU3

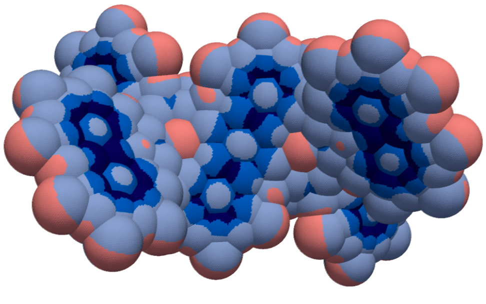

AZU4

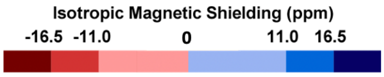

Fig. S12. 3D IMS contour maps of **AZU1**, **AZU2**, **AZU3**, and **AZU4** calculated at the B3LYP-GIAO/6-311++G**//B3LYP-D3BJ/def2-SVP level of theory.

6. Visualization procedure for .vtk files using Paraview

Preambule

- You have already installed Paraview Software: https://www.paraview.org/download/
- You have already downloaded the presets file available in the electronic supplementary information: preset_ims.json
- You have already downloaded your favorite .vtk file available in the electronic supplementary information: your_file.vtk

Open Paraview and follow the guide below.

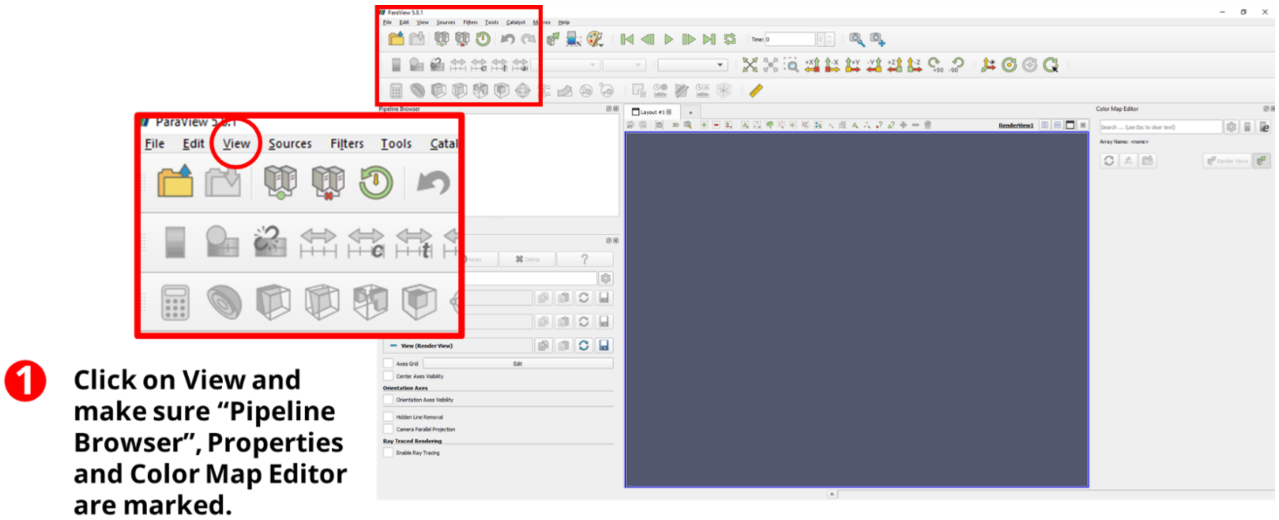

1 Click on View and make sure “Pipeline Browser”, Properties and Color Map Editor are marked.

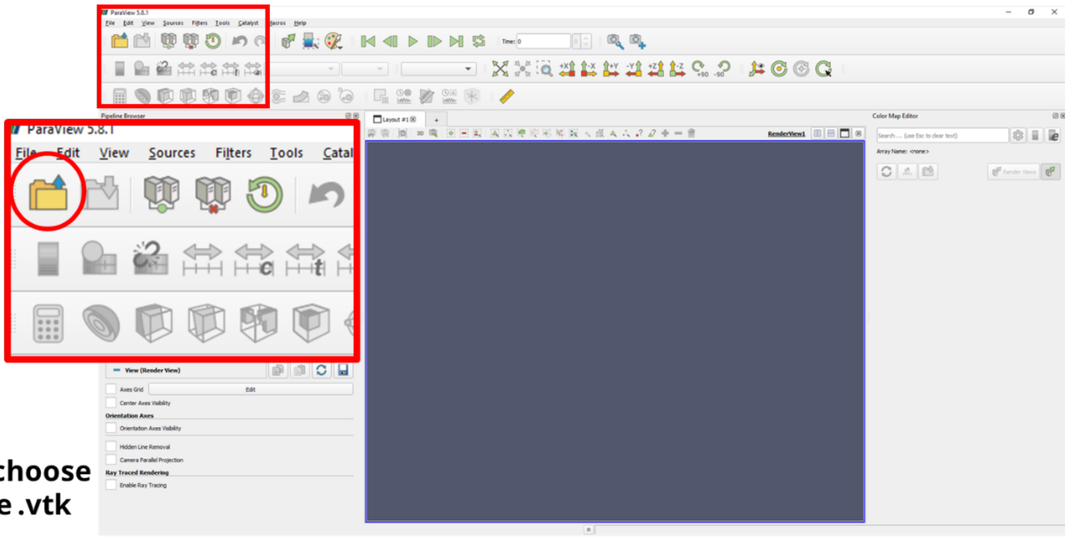

2 Open the file browser and choose your favourite .vtk file.

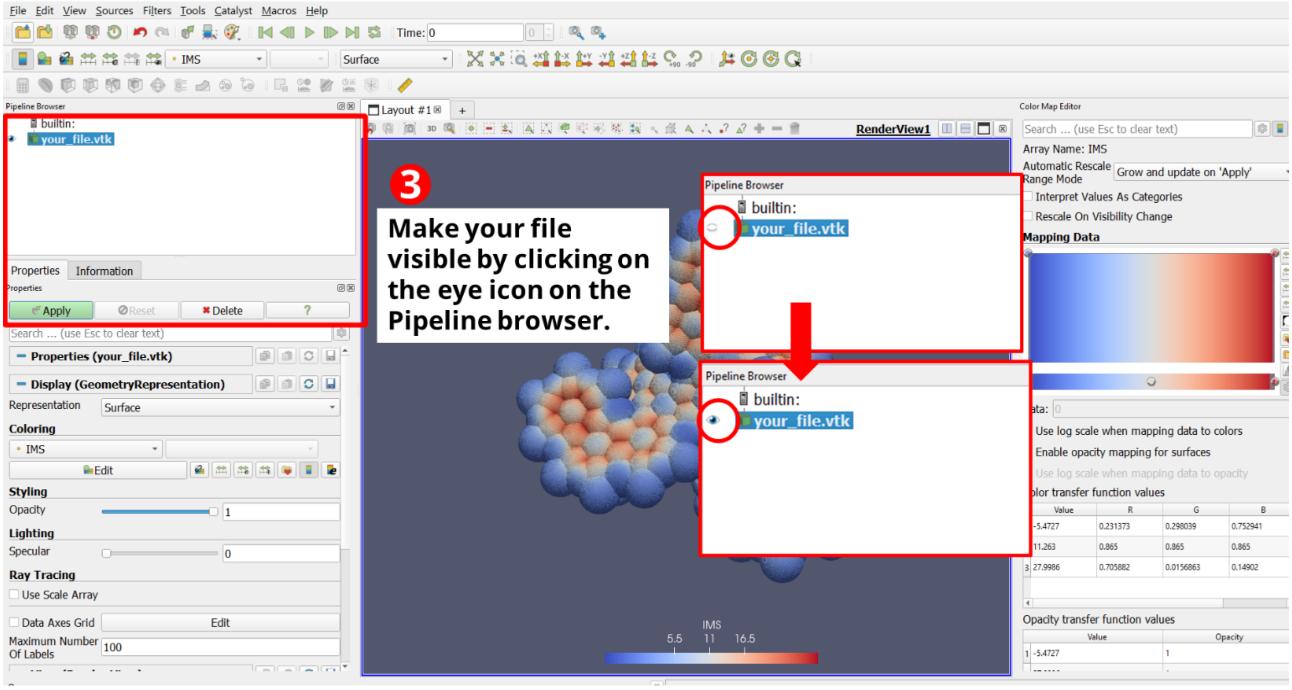
3
Make your file visible by clicking on the eye icon on the Pipeline browser.
Pipeline Browser
builtin:
your_file.vtk

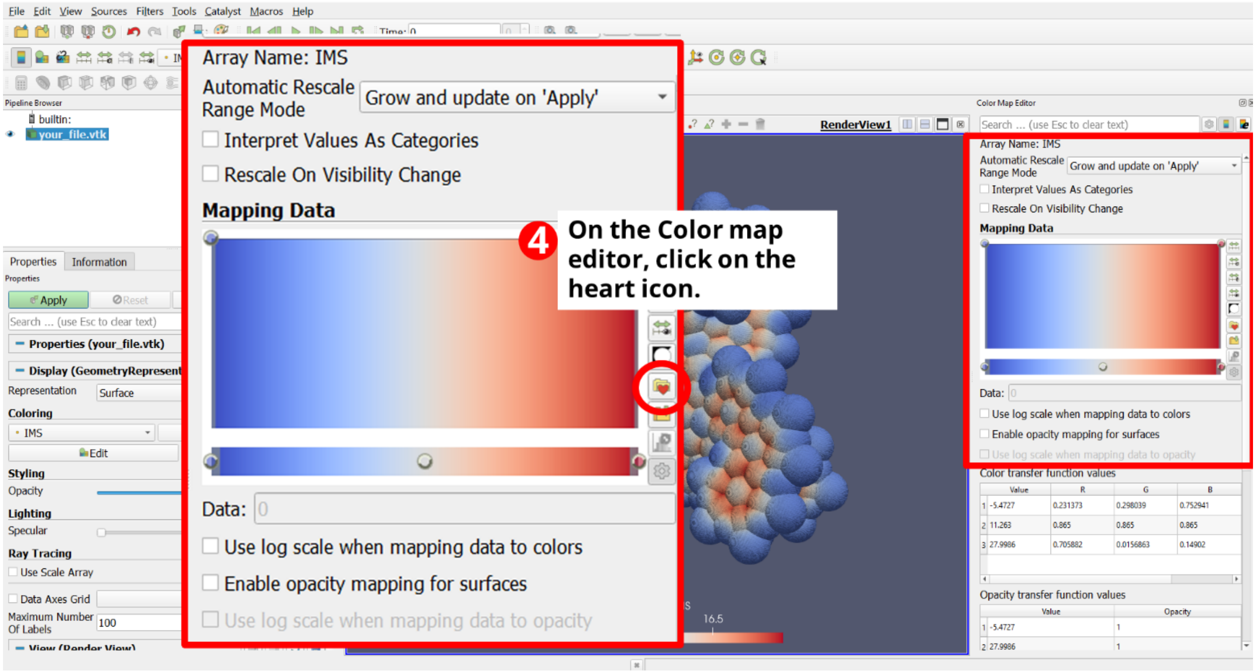
Array Name: IMS
Automatic Rescale Range Mode
Grow and update on 'Apply'
Interpret Values As Categories
Rescale On Visibility Change
Mapping Data
4
On the Color map editor, click on the heart icon.
Data:
Use log scale when mapping data to colors
Enable opacity mapping for surfaces
Use log scale when mapping data to opacity

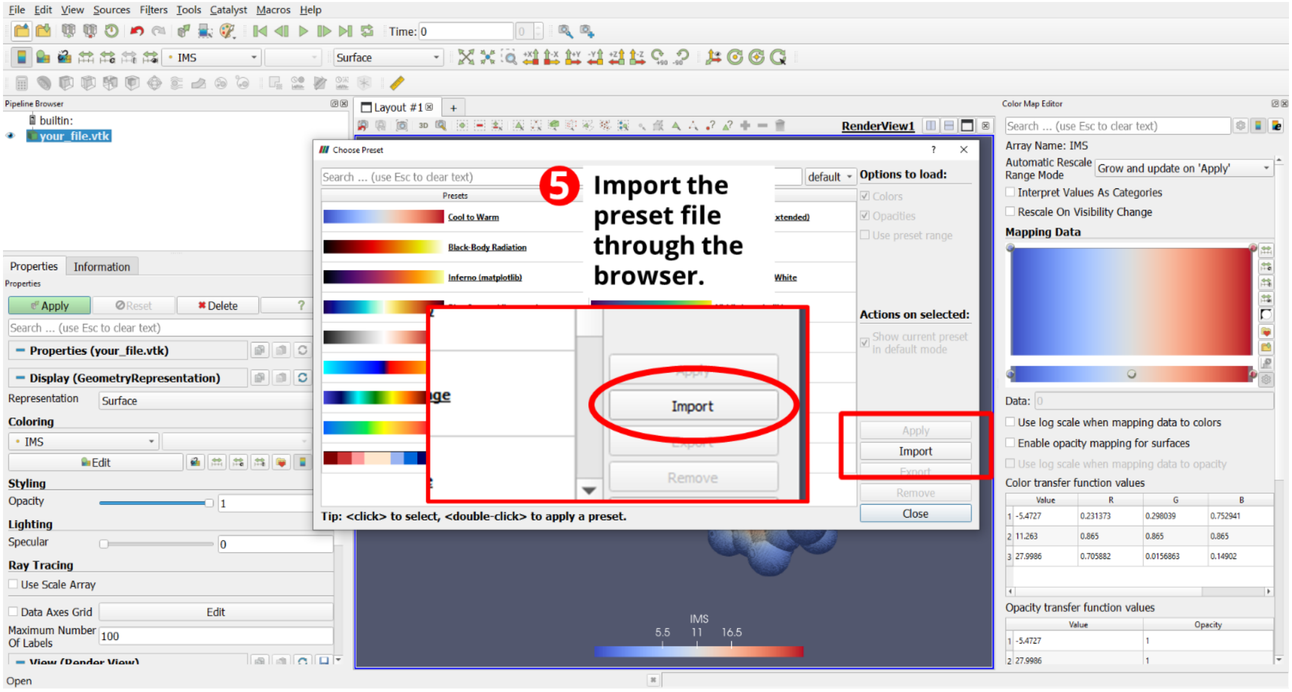
5
Import the preset file through the browser.
Choose Preset
Options to load:
Actions on selected:
Apply
Import
Export
Remove
Close
Tip: <click> to select, <double-click> to apply a preset.

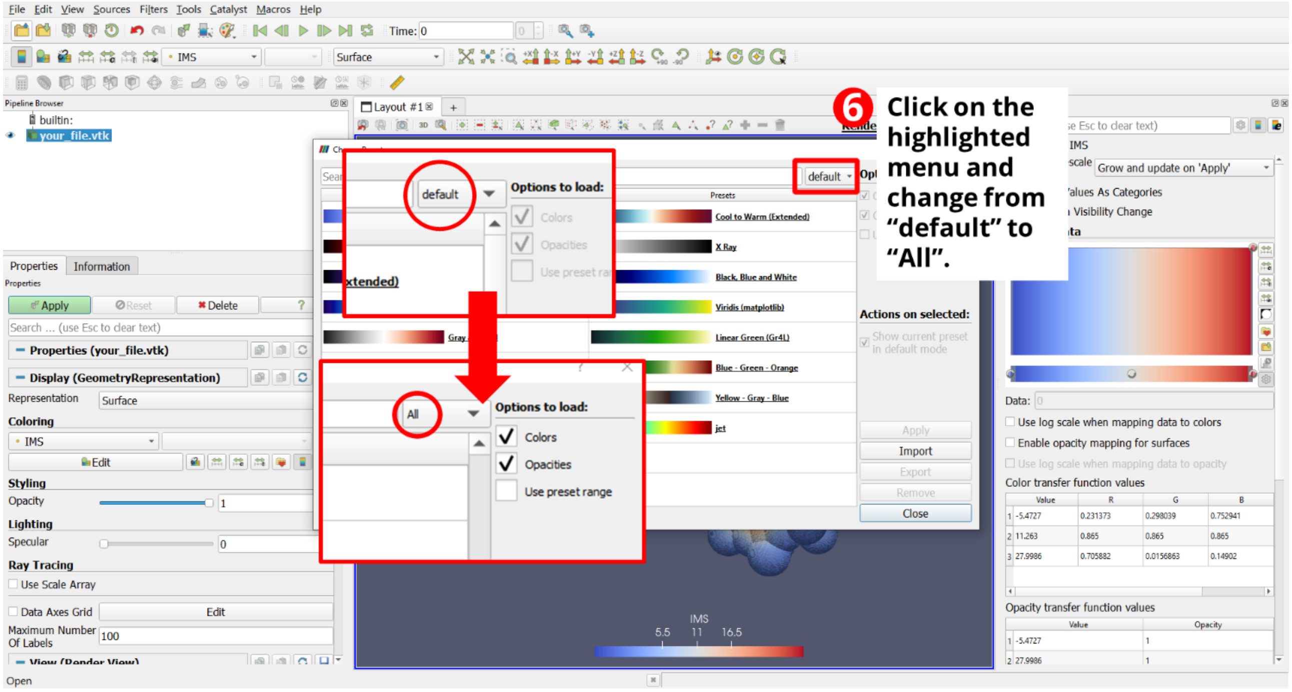
6
Click on the highlighted menu and change from “default” to “All”.
default
Options to load:
Colors
Opacities
Use preset range
All

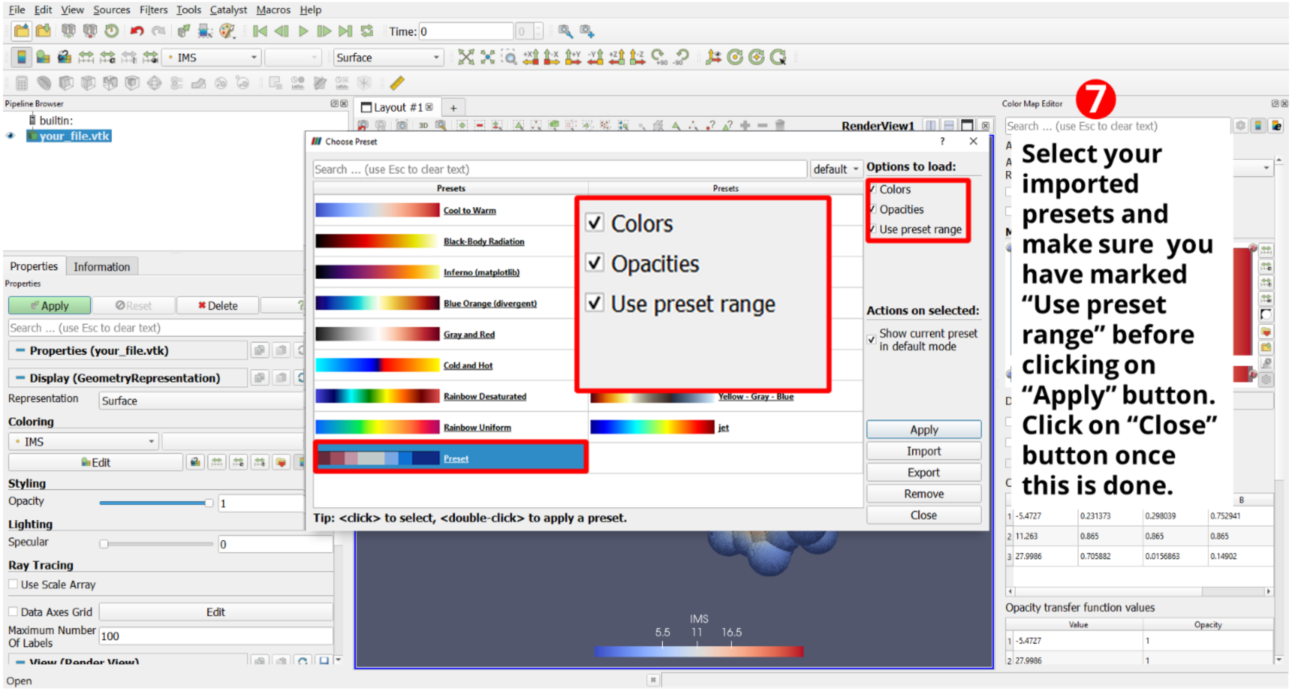
7
Select your imported presets and make sure you have marked "Use preset range" before clicking on "Apply" button. Click on "Close" button once this is done.
Choose Preset
Options to load:
Colors
Opacities
Use preset range
Actions on selected:
Show current preset in default mode
Apply
Import
Export
Remove
Close
Tip: <click> to select, <double-click> to apply a preset.

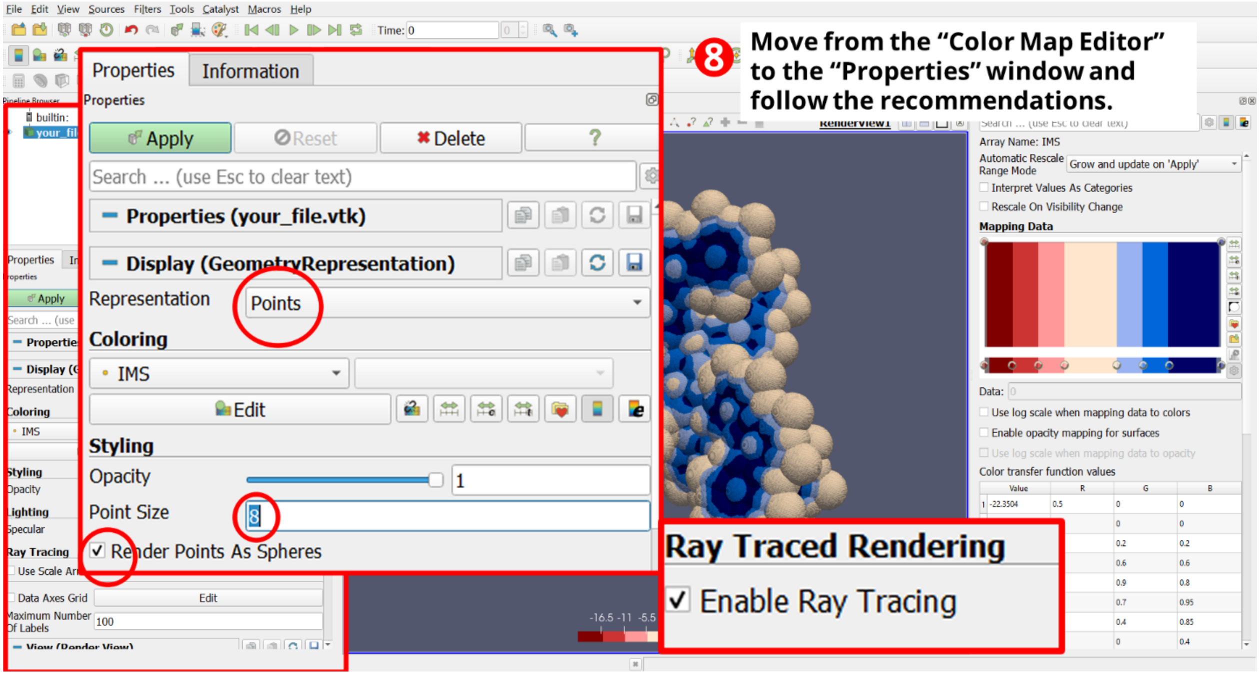
8
Move from the "Color Map Editor" to the "Properties" window and follow the recommendations.
Properties
Information
Apply
Reset
Delete
Properties (your_file.vtk)
Display (GeometryRepresentation)
Representation
Points
Coloring
IMS
Edit
Styling
Opacity
1
Point Size
8
Render Points As Spheres
Ray Traced Rendering
Enable Ray Tracing

7. References

1 M. J. Frisch, G. W. Trucks, H. B. Schlegel, G. E. Scuseria, M. A. Robb, J. R. Cheeseman, G. Scalmani, V. Barone, G. A. Petersson, H. Nakatsuji, X. Li, M. Caricato, A. V. Marenich, J. Bloino, B. G. Janesko, R. Gomperts, B. Mennucci, H. P. Hratchian, J. V. Ortiz, A. F. Izmaylov, J. L. Sonnenberg, D. Williams-Young, F. Ding, F. Lipparini, F. Egidi, J. Goings, B. Peng, A. Petrone, T. Henderson, D. Ranasinghe, V. G. Zakrzewski, J. Gao, N. Rega, G. Zheng, W. Liang, M. Hada, M. Ehara, K. Toyota, R. Fukuda, J. Hasegawa, M. Ishida, T. Nakajima, Y. Honda, O. Kitao, H. Nakai, T. Vreven, K. Throssell, J. A. Montgomery, Jr., J. E. Peralta, F. Ogliaro, M. J. Bearpark, J. J. Heyd, E. N. Brothers, K. N. Kudin, V. N. Staroverov, T. A. Keith, R. Kobayashi, J. Normand, K. Raghavachari, A. P. Rendell, J. C. Burant, S. S. Iyengar, J. Tomasi, M. Cossi, J. M. Millam, M. Klene, C. Adamo, R. Cammi, J. W. Ochterski, R. L. Martin, K. Morokuma, O. Farkas, J. B. Foresman, and D. J. Fox, Gaussian, Inc., Wallingford CT, 2016.

2 D. E. Woon and T. H. Dunning, *J. Chem. Phys.*, 1993, **98**, 1358–1371.

3 C. Lee, W. Yang and R. G. Parr, *Phys. Rev. B*, 1988, **37**, 785–789.

4 S. Grimme, J. Antony, S. Ehrlich and H. Krieg, *J. Chem. Phys.*, 2010, **132**, 154104.

5 F. Weigend and R. Ahlrichs, *Phys. Chem. Chem. Phys.*, 2005, **7**, 3297–3305.

6 K. Wolinski, J. F. Hinton and P. Pulay, *J. Am. Chem. Soc.*, 1990, **112**, 8251–8260.

7 M. J. Frisch, J. A. Pople and J. S. Binkley, *J. Chem. Phys.*, 1984, **80**, 3265–3269.

8 Chemcraft - graphical software for visualization of quantum chemistry computations. https://www.chemcraftprog.com.

9 M. Randić and A. T. Balaban, *Int. J. Quantum Chem.*, 2018, **118**, 25657.

10 M. Solà, *Front. Chem.,* 2013, **1**, 22.

11 D. Moran, F. Stahl, H. F. Bettinger, H. F. Schaefer III, P. von Ragué Schleyer, *J. Am. Chem. Soc.*, 2003, **125**, 6746–6752.

12 Y. Ruiz-Morales, J. Phys. Chem. A, 2004, **108**, 10873–10896.

13 T. Hosokawa, Y. Takahashi, T. Matsushima, S. Watanabe, S. Kikkawa, I. Azumaya, A. Tsurusaki and K. Kamikawa, *J. Am. Chem. Soc.*, 2017, **139**, 18512–18521.

14 V. Berezhnaia, M. Roy, N. Vanthuyne, M. Villa, J. V. Naubron, J. Rodriguez, Y. Coquerel and M. Gingras, *J. Am. Chem. Soc.*, 2017, **139**, 18508–18511.

15 M. Roy, V. Berezhnaia, M. Villa, N. Vanthuyne, M. Giorgi, J.-V. Naubron, S. Poyer, V. Monnier, L. Charles, Y. Carissan, D. Hagebaum-Reignier, J. Rodriguez, M. Gingras and Y. Coquerel, *Angew. Chem. Int. Ed.*, 2020, **59**, 3264–3271.
